# Supplementary material for: Correction of estimates of retention in care among a cohort of HIV-positive patients in Uganda in the period before starting ART: a sampling-based approach
Source: BMJ Open. 2018 Apr 20;8(4):e017487. doi: 10.1136/bmjopen-2017-017487 (PMC5914905; doi:10.1136/bmjopen-2017-017487)
Supplement: Author's manuscript [file bmjopen-2017-017487.draft_revisions.pdf]

BMJ Open

BMJ Open is committed to open peer review. As part of this commitment we make the peer review history of every article we publish publicly available.

When an article is published we post the peer reviewers' comments and the authors' responses online. We also post the versions of the paper that were used during peer review. These are the versions that the peer review comments apply to.

The versions of the paper that follow are the versions that were submitted during the peer review process. They are not the versions of record or the final published versions. They should not be cited or distributed as the published version of this manuscript.

BMJ Open is an open access journal and the full, final, typeset and author-corrected version of record of the manuscript is available on our site with no access controls, subscription charges or pay-per-view fees (http://bmjopen.bmj.com).

If you have any questions on BMJ Open's open peer review process please email info.bmjopen@bmj.com

BMJ Open

Correction of estimates of retention in care among a cohort of HIV-positive patients in Uganda in the period before starting ART: a sampling-based approach

Journal:	*BMJ Open*
Manuscript ID	bmjopen-2017-017487
Article Type:	Research
Date Submitted by the Author:	25-Apr-2017
Complete List of Authors:	Nyakato, Patience; London School of Hygiene and Tropical Medicine, Infectious Disease Epidemiology; Infectious Disease Institute, Makerere University<br>Kiragga, Agnes; Infectious Disease Institute, Makerere University<br>Kambugu, Andrew; Infectious Disease Institute, Makerere University<br>Bradley, John; London School of Hygiene and Tropical Medicine, Infectious Disease Epidemiology<br>Baisley, Kathy; London School of Hygiene and Tropical Medicine, Infectious Disease Epidemiology
**Primary Subject Heading**:	HIV/AIDS
Secondary Subject Heading:	Epidemiology, HIV/AIDS, Global health, Health services research
Keywords:	HIV & AIDS < INFECTIOUS DISEASES, Retention, Loss to follow up, CD4, pre-ART, Africa

SCHOLARONE™ Manuscripts

Correction of estimates of retention in care among a cohort of HIV-positive patients in Uganda in the period before starting ART: a sampling-based approach

Patience Nyakato[1,2], Agnes N Kiragga[2], Andrew Kambugu[2], John Bradley[1*], Kathy Baisley[1*]

[1]Department of Infectious Disease Epidemiology, London School of Hygiene and Tropical Medicine, London, UK; [2]Infectious Diseases Institute, College of Health Sciences, Makerere University, Kampala, Uganda

*These authors are joint senior authors on this work.

Corresponding author: Patience Nyakato; Infectious Disease Institute, Mulago Hospital Complex, College of Health Sciences, Makerere University, PO Box 22418, Kampala, Uganda. Email: pnyakato2@gmail.com Telephone: +256 787437316; Fax: +256 414 307290

Word count: 3617

For peer review only

ABSTRACT

Objective: The aim of this study was to use a sampling-based approach to obtain estimates of retention in HIV care before initiation of antiretroviral treatment (ART), corrected for outcomes in patients who were lost according to clinic registers.

Design: Retrospective cohort study of HIV positive individuals not yet eligible for ART (CD4 >500)

Setting: 3 urban and 3 rural HIV care clinics in Uganda; information was extracted from the clinic registers for all patients who had registered for pre-ART care between January–August 2015

Participants: A random sample of patients who were lost according to the clinic registers (>3 months late to scheduled visit) was traced to ascertain their outcomes.

Outcome measures: The proportion of patients lost from care was estimated using a competing risks approach, first based on the information in the clinic records alone and then using inverse probability weights to incorporate the results from tracing. Cox regression was used to determine factors associated with loss from care.

Results: Of 1153 patients registered for pre-ART care (68% female, median age 29 years, median CD4 count 645 cells/uL), 307 (27%) were lost according to clinic records. Among these, 195 (63%) were selected for tracing; outcomes were ascertained in 118 (61%). Seven patients (6%) had died, 40 (34%) were in care elsewhere, and 71 (60%) were out of care. Loss from care at 9 months was 30.2% (95% confidence interval (CI)=27.3%–33.5%). After incorporating outcomes from tracing, loss from care decreased to 18.5% (95%CI 13.8%–23.6%).

Conclusion: Estimates of loss from HIV care may be too high if based on routine clinic data alone. A sampling based approach is a feasible way of obtaining more accurate estimates of retention, accounting for transfers to other clinics.

Key words: HIV, retention in care, loss to follow up, pre-ART, CD4, Africa

For peer review only

Article Summary

Strengths and limitations of this study

- Most studies that use tracing to estimate retention in care focus on HIV-positive individuals on ART; this is one of few studies to apply these methods in the period before ART initiation.
- A sampling based approach is feasible and provides an opportunity to obtain more accurate estimates of retention in HIV care programmes in resource-limited settings
- Outcomes were not ascertained in all patients who were traced, so individuals who were traced successfully may not be representative of all who were lost.
- The follow-up time was relatively short, so some patients who were considered lost according to the clinic registers may have returned to the clinic at a later date.

For peer review only

INTRODUCTION

Access to antiretroviral therapy (ART) has expanded considerably in sub-Saharan Africa (SSA), with 12 million people in the region receiving ART in 2016.[1] With the UNAIDS 90-90-90 targets (90% of HIV positive individuals know their status, 90% of those diagnosed are on ART and 90% of those on ART are virally suppressed by 2020), more HIV positive individuals are expected to be on ART and attain viral suppression.[2-4] However, although treatment coverage in SSA doubled between 2010 and 2015, estimated coverage was still only 47% in 2015, and HIV-related mortality remains high, partly due to loss from care.[1,2]

Major gaps in HIV treatment programmes include linking individuals who test HIV positive to care, and prompt initiation of ART. Previous WHO treatment guidelines were based on CD4 count thresholds, with pre-ART care focused on immunological monitoring until individuals became eligible for ART. A 2012 systematic review of retention in HIV care in SSA found a median of 57% of individuals returned for CD4 count results after testing HIV-positive, and among those who received their results, 45% remained in care until they became eligible for ART.[5] Even among those who were ART eligible, only a median of 66% initiated ART.

The WHO released new HIV treatment guidelines in 2015, recommending that ART be offered to all HIV positive individuals irrespective of CD4 count.[6] If widely implemented, the 'treat all' approach would contribute significantly to achieving the 90-90-90 goals. As of end 2016, many countries in SSA had begun implementing the new guidelines. However, several countries had introduced the guidelines only in selected treatment sites, and others had not yet adopted the new policy.[1] Scale-up of ART treatment for all HIV positive people in resource-limited settings will require broad health systems strengthening, which in practice may mean that, for budgetary or other practical reasons, some clinics may still use CD4 counts to prioritise starting treatment. Under the 'treat all' guidelines, many individuals who are entering HIV care will have high CD4 counts and be asymptomatic, and therefore face different barriers to starting ART. Losses between testing HIV positive and ART initiation are still likely to remain. Obtaining accurate estimates of loss from care and

For peer review only

outcomes in this stage will be important for evaluating the impact of the 'treat all' guidelines, and for designing interventions to improve retention and increase the numbers starting ART.[7]

Standard estimates of retention consider those who are lost to represent disengagement from care. However, many of those lost may include transfers to other care centres.[8,9] In rural Uganda, estimates of patient retention 3 years after initiating ART increased from 60% to 85% when corrected for outcomes among those who were lost.[8,10] Therefore, estimates of retention that consider patients who are lost from care to have disengaged from care are biased, and may result in misdirection of resources at the clinic and national levels.

We used a sampling-based approach to obtain more accurate estimates of loss from pre-ART care among HIV positive individuals attending clinics in Uganda who were not yet eligible for ART. This approach involves tracing a sample of lost patients and using a weighted analysis to correct the estimates of retention in the entire clinic population, with the assumption that the traced patients are representative of all who were lost.[11-13] We also explore the effect of this approach on estimates of mortality, and factors associated with loss from care before ART initiation.

METHODS

Study setting

This was a retrospective cohort study of patients who had registered in pre-ART HIV care at 6 government clinics in Uganda: 3 urban Kampala city municipal clinics and 3 rural health centres in Hoima and Kibaale in Western Uganda. These clinics are supported by the Infectious Diseases Institute, Makerere University and offer integrated HIV testing and care facilities, provision of ART, and laboratory support. Most persons attend the HIV counselling and testing facilities as walk-ins. Individuals who test positive are referred for care within the clinic, or a preferred alternate facility, for immediate CD4 testing. Individuals are registered at the clinic at the time of initiating CD4 testing. They are then asked to return to the clinic within two weeks for their CD4 results. At the time of the study, individuals who were not

For peer review only - http://bmjopen.bmj.com/site/about/guidelines.xhtml

yet eligible for ART (CD4 count >500 cells/uL) were enrolled in a general pre-ART HIV care programme, and visited the clinic every 3 months for routine clinic check-ups and cotrimoxazole prophylaxis.

Study design

A list of all individuals who tested HIV positive between January-May 2015 (Kampala clinics) and January-August 2015 (rural clinics) and had a CD4 >500 cells/uL was obtained from the routine clinic records. Socio-demographic and routine clinical data for patients attending the Kampala clinics was extracted using the electronic patient records system (OpenMRS). For patients attending the rural clinics, the information was manually extracted from the paper-based clinic records, and entered into an Access database. Patients were then classified as either i) still in care; ii) transferred out to another clinic; iii) died; or iv) lost to follow-up, based on the information available from the clinic records. Patients were counted as lost to follow-up if they were 3 or more months late for their last scheduled visit at the clinic, and not known to have transferred out or to have died.

From the sampling frame of all patients who were classified as lost, a random sample was selected, separately for urban clinics and rural clinics, for intensive tracing. The size of the sample was based on practical considerations of how many patients could be traced at each clinic, rather than on formal sample size calculations.

Tracing was done between November 2015–March 2016 for both the urban and rural clinics. Tracers attempted to contact patients through phone calls and home visits, using addresses and locator forms containing secondary phone numbers, areas of residence, and a map to the area of residence. Patients who were successfully traced were asked to provide information about their current HIV care status: whether they were registered elsewhere and were attending another clinic; whether they had started ART elsewhere; and reasons for withdrawing from HIV care if not attending any HIV care facility. Patient deaths were ascertained through an interview with a close informant.

For peer review only - http://bmjopen.bmj.com/site/about/guidelines.xhtml

Individuals were considered to have disengaged from care if they had not registered at any other clinic and had not returned to the clinic where they were originally registered for more than 3 months after their last scheduled visit. Individuals who said they were purchasing cotrimoxazole directly from the pharmacy (i.e. not under clinician's care) or they obtained drugs through relatives/friends were also considered to have disengaged from care. Individuals who reported to have registered at another clinic were considered to be in care.

Statistical analysis

Descriptive statistics (proportions, means, medians and interquartile ranges) were used to summarise baseline characteristics. Characteristics of patients who were retained in pre-ART care and those who were lost were tabulated and compared using Chi square tests, with the Rao-Scott correction to account for correlation within clinics. In addition, characteristics of patients who were selected for tracing and traced successfully were compared with those who were selected but could not be found.

First, using only the information available from the clinic records, the proportion of patients who were lost from pre-ART care was estimated using a cumulative incidence approach, where deaths known to the clinics were treated as a competing risk. Observation time began on the date of registration at the clinic (i.e. the date of presenting for CD4 testing after HIV diagnosis) and ended at the date of known transfer out, death, loss from care (defined as 3 months after last missed appointment) or review of the clinic records (for individuals who were still in care). Patients who initiated ART were censored on the date of ART initiation. Then, a corrected analysis was conducted using the same approach but incorporating the outcomes obtained from tracing. Patients who were successfully traced were weighted using inverse probability weights, calculated as the inverse proportion of patients who were successfully traced among all patients who were lost. Patients who could not be found, or who were not selected for tracing, were given a weight of 0. Patients who were still in care according to the clinic registers were given a weight of 1. Weights were calculated separately for each clinic. For individuals who were traced and found to still be in care, observation time was considered to end at the

For peer review only - http://bmjopen.bmj.com/site/about/guidelines.xhtml

date of interview. Individuals who were traced and found to be alive but not in care were considered to have been lost 3 months after their last missed appointment at the original clinic.

A sensitivity analysis was also done: first we assumed that all individuals who were traced and not found were alive and in care elsewhere; second, we assumed that all patients who were not found were alive but not in care. Confidence intervals for the weighted estimates were obtained through bootstrapping using percentiles of the bootstrap distribution with 2000 replications.

Cox proportional hazards models were fitted to examine factors associated with loss from care, using data from the clinic registers alone and in a weighted analysis after incorporating results from tracing. Robust standard errors were used to account for correlation within clinics. Owing to the small number of covariates available, all variables were included in the final multivariate model. In the rural clinics, data on clinical covariates were often missing from the patient records; therefore, the analysis of clinical covariates was restricted to patients from the urban clinics. The appropriate functional forms of continuous covariates were explored using low order polynomials. All analyses were done using STATA version 14.2 (Stata Corp, College Station, Texas).

Ethics

Ethical approvals were obtained from the London School of Hygiene and Tropical Medicine Ethics Committee (Ref 10334), Makerere University School of Public Health Higher Degrees Research and Ethics Committee (Ref 353), Infectious Diseases Scientific Review Committee and the Uganda National Council for Science and Technology (Ref 3998). Participants who were traced successfully gave written or oral (phone interviews) informed consent.

RESULTS

Between the period of January to August 2015, 1153 individuals had registered in pre-ART care at the 6 clinics: 925 (80.2%) at the urban clinics and 228 (19.8%) at

For peer review only

For peer review only - http://bmjopen.bmj.com/site/about/guidelines.xhtml

the rural clinics. 307 (26.6%) individuals were classified as lost from care (Table 1); 207 from the urban clinics (22.4% of urban patients) and 100 from the rural clinics (43.9% of rural patients). A random sample of 195 (63.5% of those lost) patients was selected for tracing (116 from the urban clinics and 79 from the rural clinics) and 118 (60.5%) were successfully traced. 70 patients had face-to-face interviews in the clinics, 20 had telephone interviews, and 28 had home visits.

The mean (SD) age of all patients who registered in pre-ART care was 30.6 years (9.0); the majority (68.2%) were females, and the median (IQR) CD4 count was 645 (529–834) cells/uL. Characteristics of patients who were still in care were generally similar to those who were lost but there was some evidence that those who were lost were most likely to be from rural clinics and to have higher CD4 counts (Table 1). Among the 195 patients who were selected for tracing, there was no evidence of a difference in the characteristics between those who were successfully traced and those who were not found. Of those who were successfully traced, 40 (33.9%) were found to be actively in care in other clinics and 71 (60.2%) were out of care. 7 (5.9%) individuals were found to have died after having left care.

At 9 months, the cumulative incidence of loss from care based on the clinic registers was 30.2% (95% confidence interval (CI)=27.3%–33.5%; Figure 1). After incorporating outcomes from those who were successfully traced, loss from care reduced to 18.5% (95%CI 13.8%–23.6%). From the sensitivity analysis, assuming that the individuals who were traced but not found were all in care then loss was 14.9% (95%CI=10.8%–19.6%). Assuming that these patients were all out of care, then loss from care increased to 38.5% (95%CI= 31.5%–45.7%). Loss from care was higher in rural than urban clinics (46.1% vs 25.8%, respectively, based on the clinic registers). When corrected for the outcomes of those who were traced, loss from care was 28.8% (95%CI=19.9%%–37.5%) in the rural clinics and 15.3% (95%CI=9.9%–21.5%) in the urban clinics.

Based on the information available from the clinic registers alone, no patients were known to have died. After tracing, 7 patients were found to have died. After incorporating the deaths that were found through tracing, the cumulative incidence of mortality at 9 months was estimated to be 1.6% (CI=0.5%–3.0%).

For peer review only - http://bmjopen.bmj.com/site/about/guidelines.xhtml

Table 1. Characteristics of patients registered for pre-ART care

Characteristics	In care (N=864) N (col %)	Lost (N=307) N (col %)	p-value[a]	Tracked (N=195) N (col %)	Not tracked (N=112) N (col %)	p-value[b]	Found (N=118) N (col %)	Not found (N=77) N (col %)	p-value[c]
Sex			0.17			0.97			0.84
Male	258 (30.5%)	109 (35.5%)		69 (35.4%)	72 (64.3%)		41 (34.7%)	28 (36.4%)	
Female	588 (69.5%)	198 (64.5%)		126 (64.6%)	40 (35.7%)		77 (65.3%)	49 (63.6%)	
Location			0.02			0.13			0.38
Urban sites	718 (84.9%)	207 (67.4%)		116 (59.5%)	91 (81.3%)		66 (55.9%)	50 (64.9%)	
Rural sites	128 (15.1%)	100 (32.6%)		79 (40.5%)	21 (18.8%)		52 (44.1%)	27 (35.1%)	
Age in years			0.15			0.25			0.44
<20	27 (3.2 %)	18 (5.9 %)		14 (7.3 %)	4 (3.6%)		10 (8.7 %)	4 (5.3 %)	
20-29	415 (49.2%)	158 (52.1%)		96 (50.3%)	62 (55.4%)		53 (46.1%)	43 (56.6%)	
30-39	266 (31.6%)	86 (28.4%)		58 (30.4%)	28 (25%)		35 (30.4%)	23 (30.3%)	
40-49	91 (10.8%)	35 (11.6%)		22 (11.5%)	13 (11.6%)		16 (13.9%)	6 (7.9 %)	
50+	44 (5.2 %)	6 (2.0 %)		1 (0.5 %)	5 (4.5%)		1 (0.9 %)	0 (0.0 %)	
Missing	3	4		4	0		3	1	
CD4 count cell			0.08			0.07			0.27
350-499	137 (18.0%)	35 (15.6%)		21 (16.5%)	14 (14.4%)		13 (17.8%)	8 (14.8%)	
500-749	376 (49.4%)	100 (44.6%)		53 (41.7%)	47 (48.5%)		34 (46.6%)	19 (35.2%)	
750+	248 (32.6%)	89 (39.7%)		53 (41.7%)	36 (37.1%)		26 (35.6%)	27 (50.0%)	
Median (IQR)	*644 (525–812)*	*654 (540–892)*		*659 (553–918)*	*648 (534–862)*		*640 (553–885)*	*734 (565–946)*	
Missing	85	83		68	15		45	23	

[a] p value comparing those in care and lost, using Rao-Scott correction to chi squared test to account for clustered sampling. Individuals with missing values excluded from comparison. [b] p value comparing those selected for tracing and not selected, calculated as described in footnote a. [c] p value comparing those successfully traced and those not found, calculated as described in footnote a

For peer review only - http://bmjopen.bmj.com/site/about/guidelines.xhtml

In both the uncorrected and corrected analysis of factors associated with loss from care, there was strong evidence that patients from rural clinics were more likely to be lost from care than those from urban clinics (adjusted(a)HR (uncorrected)=1.95, 95%CI = 1.68–2.27, p<0.001; aHR(corrected) =2.02, 95%CI=1.49–2.73, p<0.001; Table 2). There was some evidence that older patients were less likely to be lost from care than younger patients (aHR (uncorrected) =0.79 for each 10-year increase in age, 95%CI= 0.66–0.94, p=0.007; aHR(corrected)= 0.71; 95%CI= 0.54–0.93, p=0.01). In the corrected analysis, but not in the uncorrected, there was also weak evidence that males were more likely to be lost from care.

Table 2. Factors associated with loss from care, estimated from Cox proportional hazards models, based on data in clinic registers (uncorrected) and corrected for the outcomes among patients who were successfully traced

Characteristics	Uncorrected HR[1] (95% CI)	P value	Corrected HR[1] (95% CI)	P value
Sociodemographic				
Sex		0.38		0.07
Female	1			
Male	1.17(0.82, 1.68)		1.39(0.97,1.99)	
Age per 10 years		0.007		0.01
	0.79(0.66, 0.94)		0.71(0.54,0.93)	
Location		<0.001		<0.001
Urban	1		1	
Rural	1.95(1.68, 2.27)		2.02(1.49, 2.73)	
Clinical[2]				
CD4 count (per 100 cells)		0.22		0.41
	1.04(0.98, 1.10)		1.05(0.93,1.20)	
Weight per 10kgs		<0.001		<0.001
Linear term	0.94(0.80, 1.11)		1.03(0.74, 1.42)	
Quadratic term	1.03(1.02, 1.04)		0.94(0.90, 0.98)	

[1]Sociodemographic variables adjusted for all sociodemographic variables in the table. Clinical variables adjusted for all variables in the table. [2]Analysis of associations with clinical variables restricted to urban patients.

Among patients from the urban clinics, in the uncorrected analysis, increasing weight at registration was the only clinical characteristic associated with loss from care.

For peer review only - http://bmjopen.bmj.com/site/about/guidelines.xhtml

After incorporating outcomes from the successfully tracked patients, weight at registration was still associated with loss from care, but the direction of the association had changed (Table 2).

Among the 71 patients who were successfully tracked and found not to be seeking care elsewhere, the main reasons for stopping care were that they lacked money for transport (37%), that they did not feel unwell (27%) or that they had moved to places without an HIV care facility (27%) (Table 3). Patients also reported that they lacked time (15%), purchased cotrimoxazole from other sources (14%) or did not believe that they were HIV positive (11%). The main reasons for stopping care among urban patients was not feeling unwell (41%) or having moved (39%). Among rural patients, the main reasons were lack of money for transport (50%) or that the clinic was too far away (43%)

Table 3. Reported reasons for leaving care or changing clinics among 111 patients who were traced and found alive

Reason for no longer attending clinic	No longer in care		
	Urban (N=41)	Rural (N=30)	All (N=71)
Lack money for transport	11 (26.8%)	15 (50.0%)	26 (36.6%)
Does not feel sick	17 (41.5%)	2 (6.7 %)	19 (26.8%)
Travelled/ moved away	16 (39.0%)	3 (10.0%)	19 (26.8%)
Health centre is far away	5 (12.2%)	13 (43.3%)	18 (25.4%)
Lack time	8 (19.5%)	3 (10.0%)	11 (15.5%)
Gets cotrimoxazole from other sources	7 (17.1%)	3 (10.0%)	10 (14.1%)
Doubts HIV status	4 (9.8 %)	4 (13.3%)	8 (11.3%)
Fear of being seen at the HIV clinic	0 (0.0 %)	5 (16.7%)	5 (7.0 %)
Does not like drugs/side effects	4 (9.8 %)	1 (3.3 %)	5 (7.0 %)
Using herbal/traditional medicines	0 (0.0 %)	3 (10.0%)	3 (4.2 %)
Other reason	3 (7.3 %)	2 (6.7 %)	5 (7.0 %)
Reason for changing clinics	In care at another clinic		
	Urban (N=23)	Rural (N=17)	All (N=40)
Closer to work	12 (52.2%)	6 (35.3%)	18 (45.0%)
Lack of money for transport	6 (26.1%)	4 (23.5%)	10 (25.0%)
Less waiting time	7 (30.4%)	0 (0.0 %)	7 (17.5%)
Lack time	5 (21.7%)	1 (5.9 %)	5 (12.5%)
Friends/family attend	3 (13.0%)	0 (0.0 %)	3 (7.5 %)
Fear of being seen at the first clinic	3 (13.0%)	1 (5.9 %)	3 (7.5 %)
Better service	1 (4.3 %)	1 (5.9 %)	2 (5.0 %)
Other reason	1 (4.3 %)	4 (23.5%)	5 (12.5%)

For peer review only

Among the 40 patients who reported being in care at another clinic, the main reasons for changing clinics was that the new clinic was closer to work or home (45%), they lacked money for transport (25%) or the new clinics had less waiting time (17%). The new clinic being closer was cited as the main reason for changing for both urban and rural patients (52% and 35%, respectively).

DISCUSSION

Much of the research that has been done regarding correction of estimates of retention in HIV care has concentrated on HIV positive individuals on ART. This study looked at individuals who had recently received an HIV diagnosis but were not yet eligible for ART. Based on the information from the clinic registers alone, loss from care was nearly 65% higher than after correcting for outcomes among individuals who were traced. We found that a third of the patients who were considered lost were continuing to access care at another clinic (silent transfers). We also identified deaths that had not been reported to the clinic. Other studies that have used a sampling based approach to correct estimates of retention among HIV positive individuals on ART have had similar findings.[11,14,15]

A study among HIV positive people in pre-ART care at two large clinics in Uganda in 2008–2011 found that loss from care after 2.5 years was 30.5% but decreased to 11.8% after correcting for outcomes in a sample of lost patients.[16] These figures are much lower than we found in our study in 2015, particularly in the rural clinics where corrected estimates of loss from care after 9 months were still 28.8%. The tracing period in our study was shorter and our definition of loss from care was more restrictive (3 months late to appointment vs 6 months late). Furthermore, some of the clinics in our study were smaller and more rural, so factors such as lack of transport or distance to the clinic may have presented greater barriers to retention. In the rural areas, patients often have to travel more than 10km on foot or bicycle to get to the clinics. Lastly, the CD4 threshold for ART eligibility in the earlier study was ≤350, vs ≤500 in 2015, so a larger proportion of patients in our study may have been asymptomatic and thus less motivated to remain in care.

For peer review only - http://bmjopen.bmj.com/site/about/guidelines.xhtml

Our estimates of retention, even after correction, are in line with previous studies in SSA that have shown poor retention among patients in pre-ART care. A recent review found a median of 53% of patients who had linked to pre-ART care were retained until the study endpoint.[17] Even among patients who have been identified as ART eligible, a not insignificant proportion may be lost before starting ART. A study of ART-eligible patients at a clinic in Uganda found 20% did not start ART within a year, with 8% dying whilst waiting to initiate ART.[10] Two separate reviews of retention in HIV care in SSA found that around a third of patients who were eligible for ART were lost before starting treatment.[5,18]. Factors associated with loss from care in this stage include facility-level barriers such as requirements for multiple clinic visits, inflexible clinic hours, lengthy waiting times and poor quality of care, and individual-level barriers such as fear of HIV disclosure, or limited understanding of HIV.[19]

With the new WHO ‘treat all’ guidelines, all individuals will be eligible to start ART immediately, but in practice there is likely to be a delay between linking to care after testing positive, and initiating treatment. Removing the CD4 eligibility threshold may increase the number of patients attending the clinics, which can put a strain on already overburdened health care systems. Many of the same barriers to ART initiation will remain under ‘treat all’ unless the process of starting ART is made more efficient. For successful implementation of the new guidelines, it will be essential to have accurate estimates of the proportion of people who disengage from care in the period before starting ART.

In our study, most of the reported reasons for leaving care were economic (lack of money for transport, distance from the clinic) or health systems factors (moving to a location without an HIV care facility). These factors have been commonly cited in other studies, and are a challenge to providing lifelong HIV care in resource-limited settings. A systematic review of linkage to and retention in HIV care found that transport costs and distance were two of the main barriers to retention in pre-ART care.[19] A considerable number of patients reported obtaining cotrimoxazole from other sources, presumably in response to the challenges they faced attending the clinic. Psychological factors such as feeling well, or not believing that one was HIV

For peer review only

positive, were also cited as reasons for leaving care, especially among urban patients. As has been reported in other studies, we found that younger patients were more likely to be lost from care.[19] These findings suggest that a combination of interventions may be required to improve retention in care.

We used a pragmatic approach to correct our estimates of loss from care, arithmetically upweighting the outcomes of patients who were tracked successfully to represent those of patients who were lost. Other methods have been proposed for incorporating these outcomes, including using regression models to estimate the inverse probability weights, and multiple imputation in conjunction with the ascertained outcomes. Simulations have shown that these strategies all provide less biased results than the standard uncorrected approach that is used in many epidemiological studies.[20]

Our study has several limitations. We traced only a sample of patients who were lost, and were able to find 60% of those who were selected for tracing. The individuals that we found may not have been representative of all patients who were lost. Although there was no evidence that the characteristics of those who were successfully traced were different from those who were not found, our small sample size means that we may not have had power to detect true differences if they existed, and possible bias in our estimates may still remain. In addition, we looked at loss from care over a fairly short period (9 months); it is possible that some of the individuals defined as lost based on the clinic registers would have returned to the clinic at a later date. For the individuals who were successfully traced, we relied on self-report to define whether an individual was still in care at another clinic, which may have led to over-reporting of care. Our analysis of factors associated with loss from care was limited by the small number of covariates and the large amount of missing data in the clinic databases particularly from the rural areas. Our findings from government clinics in Uganda may not be generalisable across all HIV treatment programmes in SSA, where reasons for disengagement from care, and outcomes after disengagement, may differ.

In summary, we found that estimates of loss from pre-ART care using a sampling based approach were substantially lower than those based on the clinic registers

For peer review only

alone. Retention was much lower in rural clinics than in urban clinics and was in line with previous reports of pre-ART retention in SSA. Structural factors were a key barrier to retention. These findings may have implications for the successful implementation of the ‘treat all’ guidelines, and retention in care among individuals with high CD4 counts in similar resource-limited settings.

For peer review only

Acknowledgements

We acknowledge the Kampala City Council Authority clinic staff and officials, the Infectious Disease Institute Outreach team, and the HIV care centres in Hoima and Kabaale (Dwoli health centre III, Kigorobya health centre IV and Kagadi hospital) for their support and collaboration. We thank the study participants for their participation and the project staff for their work. We acknowledge the assistance of the Statistics unit at the Infectious Disease Institute.

Competing interests

All authors have completed the ICMJE uniform disclosure form at www.icmje.org/coi_disclosure.pdf and declare: PN and JB had research grant support from EDCTP for the submitted work; KB and JB receive research grants from MRC UK and DFID; no financial relationships with any organisations that might have an interest in the submitted work in the previous three years; no other relationships or activities that could appear to have influenced the submitted work.

Funding

This work was supported by the European & Developing Countries Clinical Trials Partnership (EDCTP) through project MF.2013.40205.020; however, EDCTP cannot accept any responsibility for information or views expressed herein. JB and KB receive support from the MRC UK and DFID (MRC grant number G0700837).

Author contribution

PN, ANK, JB and KB conceived and designed the study. PN, ANK and AK conducted the study. PN analysed the data and developed the first draft. ANK, JB and KB advised on data analysis. All authors contributed to the interpretation of the data, revised the article critically and approved the final version.

For peer review only

Data sharing statement

The original data can only be accessed by the research investigators at the Infectious Disease Institute. PN can be contacted if anonymised data or the statistical code are required.

For peer review only

REFERENCES

1. World Health Organization. Progress report 2016: prevent HIV, test and treat all: WHO support for country impact. Geneva, Switzerland; 2016.
2. Stover J, Bollinger L, Izazola JA, et al., What is required to end the AIDS epidemic as a public health threat by 2030? The cost and impact of the fast-track approach. *PloS ONE* 2016;11(5):e0154893.
3. UNAIDS. 90-90-90 An ambitious treatment target to help end the AIDS epidemic. Geneva, Switzerland 2014. Available from http://www.unaids.org/en/resources/documents/2017/90-90-90.
4. Kisesa A, Chamla D. Getting to 90–90–90 targets for children and adolescents HIV in low and concentrated epidemics: bottlenecks, opportunities, and solutions. *Curr Opin HIV AIDS* 2016;11(Suppl1):S1.
5. Kranzer K, Govindasamy D, Ford N, et al. Quantifying and addressing losses along the continuum of care for people living with HIV infection in sub-Saharan Africa: a systematic review. *J Int AIDS Soc* 2012;15(2):17383
6. World Health Organization. Consolidated guidelines on the use of antiretroviral drugs for treating and preventing HIV infection. Recommendations for a public health approach. Second edition. Geneva, Switzerland; 2016.
7. Fox M, Rosen A. new cascade of HIV care for the era of ‘treat all’. *PLoS Medicine* 2017;14(4):e1002268.
8. Geng EH, Glidden DV, Bwana MB, et al. Retention in care and connection to care among HIV-infected patients on antiretroviral therapy in Africa: estimation via a sampling-based approach. *PLoS ONE* 2011;6(7):e21797.
9. Tweya H, Feldacker C, Estill J, et al. Are they really lost? ‘True’ status and reasons for treatment discontinuation among HIV infected patients on antiretroviral therapy considered lost to follow up in urban Malawi. *PLoS ONE* 2013;8(9):e75761.
10. Geng EH, Bwana MB, Muyindike W, et al. Failure to initiate antiretroviral therapy, loss to follow-up and mortality among HIV-infected patients during the pre-ART period in Uganda. *J Acquir Immune Defic Syndr* 2013;63(2):e64-71.

For peer review only

11. Geng EH, Glidden DV, Emenyonu N, et al. Tracking a sample of patients lost to follow-up has a major impact on understanding determinants of survival in HIV-infected patients on antiretroviral therapy in Africa. *Trop Med Int Health*, 2010;15(Suppl1):63-69.

12. Geng EH, Emenyonu N, Bwana MB, et al. Sampling-based approach to determining outcomes of patients lost to follow-up in antiretroviral therapy scale-up programs in Africa. *JAMA* 2008;300(5):506-507.

13. Geng EH, Odeny TA, Lyamuya R, et al. Retention in care and patient-reported reasons for undocumented transfer or stopping care among HIV-infected patients on antiretroviral therapy in Eastern Africa: application of a sampling-based approach. *Clin Infect Dis* 2015:civ1004.

14. Kiragga AN, Castelnuovo B, Musomba R, et al. Comparison of methods for correction of mortality estimates for loss to follow-up after ART initiation: a case of the Infectious Diseases Institute, Uganda. *PLoS ONE* 2013;8(12):e83524.

15. Egger M, Spycher BD, Sidle J, et al. Correcting mortality for loss to follow-up: a nomogram applied to antiretroviral treatment programmes in sub-Saharan Africa. *PLoS Med*, 2011;8(1):e1000390.

16. Namusobya J, Semitala FC, Amanyire G, et al. High retention in care among HIV-infected patients entering care with CD4 levels >350 cells/uL under routine program conditions in Uganda. *Clin Infect Dis* 2013;57(9):1343-1350.

17. Plazy M, Orne-Gliemann J, Dabis F, et al. Retention in care prior to antiretroviral treatment eligibility in sub-Saharan Africa: a systematic review of the literature. *BMJ Open* 2015;5(6):e006927.

18. Rosen S, Fox MP. Retention in HIV care between testing and treatment in sub-Saharan Africa: a systematic review. *PLoS Med* 2011;8(7):e1001056.

19. Govindasamy D, Ford N, Kranzer K. Risk factors, barriers and facilitators for linkage to antiretroviral therapy care: a systematic review. *AIDS* 2012;26(16):2059-2067.

20. Schomaker M, Gsponer T, Estill J, et al. Non-ignorable loss to follow-up: correcting mortality estimates based on additional outcome ascertainment. *Stat Med* 2014;33(1):129-142.

For peer review only

Figure 1: Uncorrected and corrected cumulative incidence of loss from care among patients with CD4 >500 registered for HIV care at 6 clinics

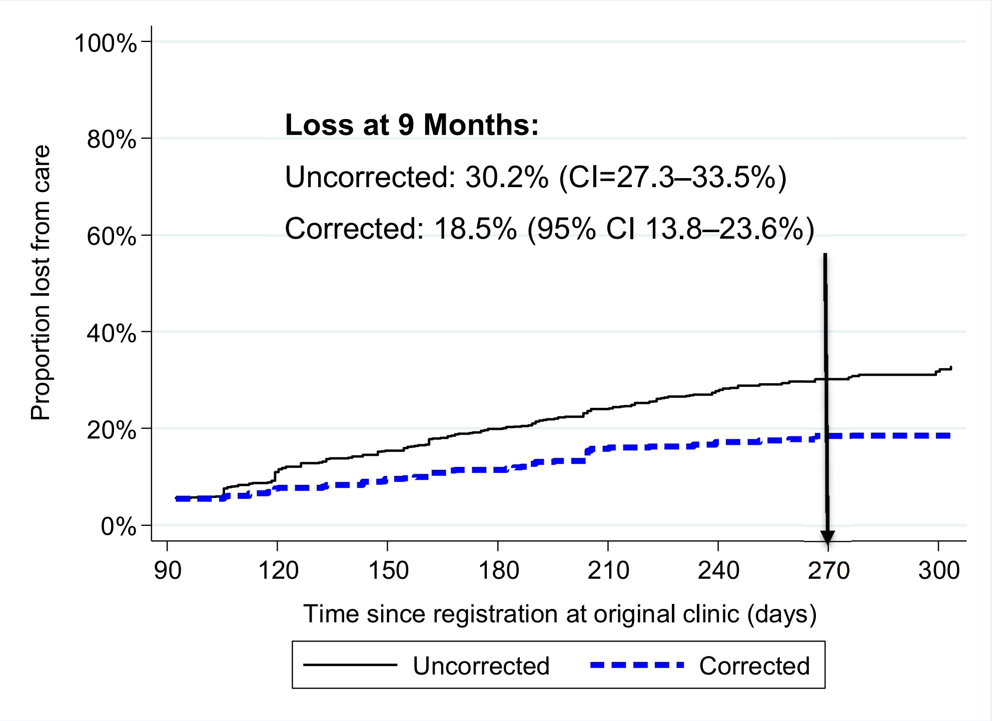

review only

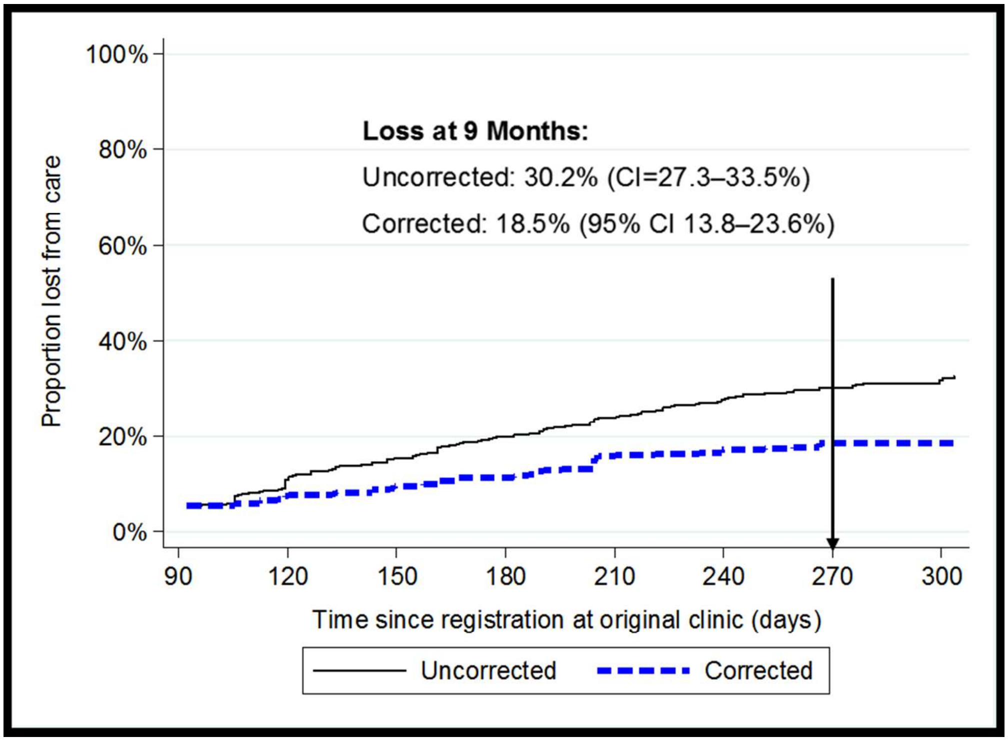

Figure 1: Uncorrected and corrected cumulative incidence of loss from care among patients with CD4 >500 registered for HIV care at 6 clinics

78x57mm (300 x 300 DPI)

STROBE 2007 (v4) checklist of items to be included in reports of observational studies in epidemiology*
Checklist for cohort, case-control, and cross-sectional studies (combined)

Section/Topic	Item #	Recommendation	Reported on page #
**Title and abstract**	1	(*a*) Indicate the study's design with a commonly used term in the title or the abstract	1
		(*b*) Provide in the abstract an informative and balanced summary of what was done and what was found	2
**Introduction**			
Background/rationale	2	Explain the scientific background and rationale for the investigation being reported	4
Objectives	3	State specific objectives, including any pre-specified hypotheses	5
**Methods**			
Study design	4	Present key elements of study design early in the paper	5, 6
Setting	5	Describe the setting, locations, and relevant dates, including periods of recruitment, exposure, follow-up, and data collection	5, 6
Participants	6	(*a*) *Cohort study*—Give the eligibility criteria, and the sources and methods of selection of participants. Describe methods of follow-up<br>*Case-control study*—Give the eligibility criteria, and the sources and methods of case ascertainment and control selection. Give the rationale for the choice of cases and controls<br>*Cross-sectional study*—Give the eligibility criteria, and the sources and methods of selection of participants	6,7
		(*b*) *Cohort study*—For matched studies, give matching criteria and number of exposed and unexposed<br>*Case-control study*—For matched studies, give matching criteria and the number of controls per case	
Variables	7	Clearly define all outcomes, exposures, predictors, potential confounders, and effect modifiers. Give diagnostic criteria, if applicable	6-8
Data sources/ measurement	8*	For each variable of interest, give sources of data and details of methods of assessment (measurement). Describe comparability of assessment methods if there is more than one group	6, 7
Bias	9	Describe any efforts to address potential sources of bias	7-8
Study size	10	Explain how the study size was arrived at	6
Quantitative variables	11	Explain how quantitative variables were handled in the analyses. If applicable, describe which groupings were chosen and why	7-8
Statistical methods	12	(*a*) Describe all statistical methods, including those used to control for confounding	7-8
		(*b*) Describe any methods used to examine subgroups and interactions	7-8
		(*c*) Explain how missing data were addressed	8
		(*d*) *Cohort study*—If applicable, explain how loss to follow-up was addressed<br>*Case-control study*—If applicable, explain how matching of cases and controls was addressed	8

For peer review only

		Cross-sectional study—If applicable, describe analytical methods taking account of sampling strategy8	
		(*e*) Describe any sensitivity analyses	8
**Results**			
Participants	13*	(a) Report numbers of individuals at each stage of study—eg numbers potentially eligible, examined for eligibility, confirmed eligible, included in the study, completing follow-up, and analysed	8-9
		(b) Give reasons for non-participation at each stage	9
		(c) Consider use of a flow diagram	N/A
Descriptive data	14*	(a) Give characteristics of study participants (eg demographic, clinical, social) and information on exposures and potential confounders	9
		(b) Indicate number of participants with missing data for each variable of interest	10
		(c) *Cohort study*—Summarise follow-up time (eg, average and total amount)	
Outcome data	15*	*Cohort study*—Report numbers of outcome events or summary measures over time	9, 11
		Case-control study—Report numbers in each exposure category, or summary measures of exposure	
		Cross-sectional study—Report numbers of outcome events or summary measures	
Main results	16	(*a*) Give unadjusted estimates and, if applicable, confounder-adjusted estimates and their precision (eg, 95% confidence interval). Make clear which confounders were adjusted for and why they were included	9, 11, 12
		(*b*) Report category boundaries when continuous variables were categorized	N/A
		(*c*) If relevant, consider translating estimates of relative risk into absolute risk for a meaningful time period	N/A
Other analyses	17	Report other analyses done—eg analyses of subgroups and interactions, and sensitivity analyses	9, 11
**Discussion**			
Key results	18	Summarise key results with reference to study objectives	13
Limitations	19	Discuss limitations of the study, taking into account sources of potential bias or imprecision. Discuss both direction and magnitude of any potential bias	15
Interpretation	20	Give a cautious overall interpretation of results considering objectives, limitations, multiplicity of analyses, results from similar studies, and other relevant evidence	15,16
Generalisability	21	Discuss the generalisability (external validity) of the study results	15
**Other information**			
Funding	22	Give the source of funding and the role of the funders for the present study and, if applicable, for the original study on which the present article is based	17

*Give information separately for cases and controls in case-control studies and, if applicable, for exposed and unexposed groups in cohort and cross-sectional studies.

Note: An Explanation and Elaboration article discusses each checklist item and gives methodological background and published examples of transparent reporting. The STROBE checklist is best used in conjunction with this article (freely available on the Web sites of PLoS Medicine at http://www.plosmedicine.org/, Annals of Internal Medicine at http://www.annals.org/, and Epidemiology at http://www.epidem.com/). Information on the STROBE Initiative is available at www.strobe-statement.org.

For peer review only

BMJ Open

Correction of estimates of retention in care among a cohort of HIV-positive patients in Uganda in the period before starting ART: a sampling-based approach

Journal:	*BMJ Open*
Manuscript ID	bmjopen-2017-017487.R1
Article Type:	Research
Date Submitted by the Author:	05-Dec-2017
Complete List of Authors:	Nyakato, Patience; London School of Hygiene and Tropical Medicine, Infectious Disease Epidemiology; Infectious Disease Institute, Makerere University<br>Kiragga, Agnes; Infectious Disease Institute, Makerere University<br>Kambugu, Andrew; Infectious Disease Institute, Makerere University<br>Bradley, John; London School of Hygiene and Tropical Medicine, MRC Tropical Epidemiology Group, Infectious Disease Epidemiology<br>Baisley, Kathy; London School of Hygiene and Tropical Medicine, MRC Tropical Epidemiology Group, Infectious Disease Epidemiology
**Primary Subject Heading**:	HIV/AIDS
Secondary Subject Heading:	Epidemiology, HIV/AIDS, Global health, Health services research
Keywords:	HIV & AIDS < INFECTIOUS DISEASES, Retention, Loss to follow up, CD4, pre-ART, Africa

SCHOLARONE™ Manuscripts

Correction of estimates of retention in care among a cohort of HIV-positive patients in Uganda in the period before starting ART: a sampling-based approach

Patience Nyakato[1,2], Agnes N Kiragga[2], Andrew Kambugu[2], John Bradley[1*], Kathy Baisley[1*]

[1]MRC Tropical Epidemiology Group, Department of Infectious Disease Epidemiology, London School of Hygiene and Tropical Medicine, London, UK; [2]Infectious Diseases Institute, College of Health Sciences, Makerere University, Kampala, Uganda

*These authors are joint senior authors on this work.

Corresponding author: Patience Nyakato; Infectious Disease Institute, Mulago Hospital Complex, College of Health Sciences, Makerere University, PO Box 22418, Kampala, Uganda. Email: pnyakato2@gmail.com Telephone: +256 787437316; Fax: +256 414 307290

Word count: 4314

For peer review only

ABSTRACT

Objective: The aim of this study was to use a sampling-based approach to obtain estimates of retention in HIV care before initiation of antiretroviral treatment (ART), corrected for outcomes in patients who were lost according to clinic registers.

Design: Retrospective cohort study of HIV positive individuals not yet eligible for ART (CD4 >500)

Setting: 3 urban and 3 rural HIV care clinics in Uganda; information was extracted from the clinic registers for all patients who had registered for pre-ART care between January–August 2015

Participants: A random sample of patients who were lost according to the clinic registers (>3 months late to scheduled visit) was traced to ascertain their outcomes.

Outcome measures: The proportion of patients lost from care was estimated using a competing risks approach, first based on the information in the clinic records alone and then using inverse probability weights to incorporate the results from tracing. Cox regression was used to determine factors associated with loss from care.

Results: Of 1153 patients registered for pre-ART care (68% female, median age 29 years, median CD4 count 645 cells/uL), 307 (27%) were lost according to clinic records. Among these, 195 (63%) were selected for tracing; outcomes were ascertained in 118 (61%). Seven patients (6%) had died, 40 (34%) were in care elsewhere, and 71 (60%) were out of care. Loss from care at 9 months was 30.2% (95% confidence interval (CI)=27.3%–33.5%). After incorporating outcomes from tracing, loss from care decreased to 18.5% (95%CI 13.8%–23.6%).

Conclusion: Estimates of loss from HIV care may be too high if based on routine clinic data alone. A sampling based approach is a feasible way of obtaining more accurate estimates of retention, accounting for transfers to other clinics.

Key words: HIV, retention in care, loss to follow up, pre-ART, CD4, Africa

For peer review only

Article Summary

Strengths and limitations of this study

- Most studies that use tracing to estimate retention in care focus on HIV-positive individuals on ART; this is one of few studies to apply these methods in the period before ART initiation.
- A sampling based approach is feasible and provides an opportunity to obtain more accurate estimates of retention in HIV care programmes in resource-limited settings
- Outcomes were not ascertained in all patients who were traced, so individuals who were traced successfully may not be representative of all who were lost.
- The follow-up time was relatively short, so some patients who were considered lost according to the clinic registers may have returned to the clinic at a later date.

For peer review only

INTRODUCTION

Access to antiretroviral therapy (ART) has expanded considerably in sub-Saharan Africa (SSA), with 12 million people in the region receiving ART in 2016.[1] With the UNAIDS 90-90-90 targets (90% of HIV positive individuals know their status, 90% of those diagnosed are on ART and 90% of those on ART are virally suppressed by 2020), more HIV positive individuals are expected to be on ART and attain viral suppression.[2-4] However, although treatment coverage in SSA doubled between 2010 and 2015, estimated coverage was still only 47% in 2015, and HIV-related mortality remains high, partly due to loss from care.[1,2]

Major gaps in HIV treatment programmes include linking individuals who test HIV positive to care, and prompt initiation of ART. Previous WHO treatment guidelines were based on CD4 count thresholds, with pre-ART care focused on immunological monitoring until individuals became eligible for ART. A 2012 systematic review of retention in HIV care in SSA found a median of 57% of individuals returned for CD4 count results after testing HIV-positive, and among those who received their results, 45% remained in care until they became eligible for ART.[5] Even among those who were ART eligible, only a median of 66% initiated ART[5].

The WHO released new HIV treatment guidelines in 2015, recommending that ART be offered to all HIV positive individuals irrespective of CD4 count.[6] If widely implemented, the 'treat all' approach would contribute significantly to achieving the 90-90-90 goals. As of end 2016, many countries in SSA had begun implementing the new guidelines. However, several countries had introduced the guidelines only in selected treatment sites, and others had not yet adopted the new policy.[1] Scale-up of ART treatment for all HIV positive people in resource-limited settings will require broad health systems strengthening, which in practice may mean that, for budgetary or other practical reasons, some clinics may still use CD4 counts to prioritise starting treatment. Uganda officially rolled out the test and treat guidelines in November 2016. By end 2017, nearly all government clinics had implemented test and treat. However, in practice, priority for ART initiation is given to existing patients in pre-ART care. Furthermore, ART 'stock outs' are still common, so individuals who

For peer review only

are newly diagnosed are likely to have some period of pre-ART care. In March 2017, an estimated 6% of HIV positive persons who were enrolled in HIV care were not on ART.[7] In addition, 48% of men who had tested HIV positive have not yet initiated ART.[7]

Under the 'treat all' guidelines, many individuals who are entering HIV care will have high CD4 counts and be asymptomatic, and therefore face different barriers to starting ART. Losses between testing HIV positive and ART initiation are still likely to remain. Obtaining accurate estimates of loss from care and outcomes in this stage will be important for evaluating the impact of the 'treat all' guidelines, and for designing interventions to improve retention and increase the numbers starting ART.[8]

Standard estimates of retention consider those who are lost to represent disengagement from care. However, many of those lost may include transfers to other care centres.[9,10] In rural Uganda, estimates of patient retention 3 years after initiating ART increased from 60% to 85% when corrected for outcomes among those who were lost.[9,11] Therefore, estimates of retention that consider patients who are lost from care to have disengaged from care are biased, and may result in misdirection of resources at the clinic and national levels.

We used a sampling-based approach to obtain more accurate estimates of loss from pre-ART care among HIV positive individuals attending clinics in Uganda who were not yet eligible for ART. This approach involves tracing a sample of lost patients and using a weighted analysis to correct the estimates of retention in the entire clinic population, with the assumption that the traced patients are representative of all who were lost.[12-14] We also explore the effect of this approach on estimates of mortality, and factors associated with loss from care before ART initiation.

For peer review only

METHODS

Study setting

This was a retrospective cohort study of patients who had registered in pre-ART HIV care at 6 government clinics in Uganda: 3 urban Kampala city municipal clinics (Kisenyi, Kawala and Kitebi) which are managed by the Kampala City Council Authority (KCCA), and 3 rural health centres in Hoima and Kibaale districts in western Uganda, which are run by the Uganda Ministry of Health. The 3 urban clinics are level IV health centres, which have a target catchment population of 100,000. Kisenyi is the largest of these, and serves a particularly economically impoverished area. The rural facilities included one level IV (Kiogorobya) and one level III (Dwoli) health centre in Hoima district, and Kagadi District Hospital in Kibaale district. Hoima district had an estimated population of 574,000 in the 2014 census, and Kibaale had an estimated population of 789,000.

All 6 facilities are supported by the Infectious Diseases Institute (IDI), Makerere University and offer integrated HIV testing and care facilities, provision of ART, and laboratory support. Most persons attend the HIV counselling and testing facilities as walk-ins. Individuals who test positive are referred for care within the clinic, or a preferred alternate facility, for immediate CD4 testing. Individuals are registered at the clinic at the time of initiating CD4 testing. They are then asked to return to the clinic within two weeks for their CD4 results. At the time of the study (2015), individuals who were not yet eligible for ART were enrolled in a general pre-ART HIV care programme, and visited the clinic every 3 months for routine clinic check-ups and cotrimoxazole prophylaxis. In 2015, the CD4 count threshold for ART initiation was ≤500 cells/uL; however, in practice, priority was given to individuals with CD4 counts <350. The 3 Kampala clinics are among 7 KCCA clinics supported by IDI; in June 2015, there were 33,514 HIV positive persons receiving HIV care services in these facilities, of whom 87% were on ART[15]

Study design

A list of all individuals who tested HIV positive between January-May 2015 (Kampala clinics) and January-August 2015 (rural clinics) and were not yet eligible for ART was

For peer review only - http://bmjopen.bmj.com/site/about/guidelines.xhtml

obtained from the routine clinic records. Socio-demographic and routine clinical data for patients attending the Kampala clinics was extracted using the electronic patient records system (OpenMRS). For patients attending the rural clinics, the information was manually extracted from the paper-based clinic records, and entered into an Access database. Patients were then classified as either i) still in care; ii) transferred out to another clinic; iii) died; or iv) lost to follow-up, based on the information available from the clinic records. Patients were counted as lost to follow-up if they were 3 or more months late for their last scheduled visit at the clinic, and not known to have transferred out or to have died.

From the sampling frame of all patients who were classified as lost, a random sample was selected, separately for urban clinics and rural clinics, for intensive tracing. The size of the sample was based on practical considerations of how many patients could be traced at each clinic with available resources, rather than on formal sample size calculations.

Tracing was done between November 2015–March 2016 in both the urban and rural clinics. Tracers attempted to contact patients through phone calls and home visits, using addresses and locator forms containing secondary phone numbers, areas of residence, and a map to the area of residence. For patients who could not be contacted through phone calls, home visits using locator forms were used; at least 3 visit attempts were made before declaring the patient unreachable.

Patients who were successfully traced were asked to provide information about their current HIV care status: whether they were registered elsewhere and were attending another clinic; whether they had started ART elsewhere; and reasons for withdrawing from HIV care if not attending any HIV care facility. Patient deaths were ascertained through an interview with a close informant.

Individuals were considered to have disengaged from care if they had not registered at any other clinic and had not returned to the clinic where they were originally registered for more than 3 months after their last scheduled visit. Individuals who said they were purchasing cotrimoxazole directly from the pharmacy (i.e. not under clinician's care) or they obtained drugs through relatives/friends were also

For peer review only

considered to have disengaged from care. Individuals who reported to have registered at another clinic were asked the name of the other clinic, the date of their next appointment, and for evidence of registration such as a patient card with a current appointment date. Those who had a current patient card, or gave a valid name of an HIV care clinic with a current appointment date, were considered to be in care.

Interviews were conducted by trained nurse counsellors, all of whom had previous research experience. Information was collected using a standard structured questionnaire. The nurse counsellors made the contact attempts by telephone, and traced urban patients at their homes if the information on the locator form was sufficient. Many of the rural patients could not be reached by telephone, and the information on the locator forms was inadequate. Therefore, in the rural clinics, ‘expert’ patients from each clinic were paired with the nurse interviewers to help trace patients in the community. Expert patients also helped with tracing in the urban clinics, for patients who could not be found through the locator forms. Expert patients serve as community volunteers at the clinic, and offer support to fellow patients in HIV care. They are familiar with the surrounding community and are often called on by the clinic to help trace persons on ART who have missed their visits.

For peer review only

Statistical analysis

Descriptive statistics (proportions, means, medians and interquartile ranges) were used to summarise baseline characteristics. Characteristics of patients who were retained in pre-ART care and those who were lost were tabulated and compared using Chi square tests, with the Rao-Scott correction to account for correlation within clinics. In addition, characteristics of patients who were selected for tracing and traced successfully were compared with those who were selected but could not be found.

First, using only the information available from the clinic records, the proportion of patients who were lost from pre-ART care was estimated using a cumulative incidence approach, where deaths known to the clinics were treated as a competing risk. Observation time began on the date of registration at the clinic (i.e. the date of

For peer review only - http://bmjopen.bmj.com/site/about/guidelines.xhtml

presenting for CD4 testing after HIV diagnosis) and ended at the earliest of the date of known transfer out, death, loss from care (defined as 3 months after last missed appointment) or review of the clinic records (for individuals who were still in care). Patients who initiated ART were censored on the date of ART initiation. Then, a corrected analysis was conducted using the same approach but incorporating the outcomes obtained from tracing. The outcomes of patients who were successfully traced were weighted using inverse probability weights, calculated as the inverse proportion of patients who were successfully traced among all patients who were lost. Patients who could not be found, or who were not selected for tracing, were given a weight of 0. Patients who were still in care according to the clinic registers were given a weight of 1. Weights were calculated separately for each clinic. For example, in a clinic with 100 patients of whom 30 were lost, 10 were successfully traced and 6 were found to be still in care, the weights for the patients who were traced would be 30/10=3. The corrected estimate for the proportion in care would be calculated as: [70 x 1 + 6 (found to be still in care) x 3] / [70 x 1 + 6 x 3 + 4 (found to be out of care) x 3] = 88/100. For individuals who were traced and found to still be in care, observation time was considered to end at the date of interview. Individuals who were traced and found to be alive but not in care were considered to have been lost 3 months after their last missed appointment at the original clinic.

A sensitivity analysis was also done: first we assumed that all individuals who were traced and not found were alive and in care elsewhere; second, we assumed that all patients who were not found were alive but not in care. Confidence intervals for the weighted estimates were obtained through bootstrapping using percentiles of the bootstrap distribution with 2000 replications.

Cox proportional hazards models were fitted to examine factors associated with loss from care, using data from the clinic registers alone and in a weighted analysis after incorporating results from tracing. Robust standard errors were used to account for correlation within clinics. Owing to the small number of covariates available, all variables were included in the final multivariate model. In the rural clinics, data on clinical covariates were often missing from the patient records; therefore, the analysis of clinical covariates was restricted to patients from the urban clinics. The appropriate functional forms of continuous covariates were explored using low order

For peer review only - http://bmjopen.bmj.com/site/about/guidelines.xhtml

polynomials (quadratic and cubic forms). All analyses were done using STATA version 14.2 (Stata Corp, College Station, Texas).

Ethics

Ethical approvals were obtained from the London School of Hygiene and Tropical Medicine Ethics Committee (Ref 10334), Makerere University School of Public Health Higher Degrees Research and Ethics Committee (Ref 353), Infectious Diseases Scientific Review Committee and the Uganda National Council for Science and Technology (Ref 3998). Patients give informed consent at the time of registration in HIV care at the clinics, to be traced in case they miss their appointments. Patients who were traced successfully gave additional written or oral (phone interviews) informed consent for participation in the current study.

RESULTS

Between the period of January to August 2015, 1153 individuals had registered in pre-ART care at the 6 clinics: 925 (80.2%) at the urban clinics and 228 (19.8%) at the rural clinics. 307 (26.6%) individuals were classified as lost from care (Table 1); 207 from the urban clinics (22.4% of urban patients) and 100 from the rural clinics (43.9% of rural patients). A random sample of 195 (63.5% of those lost) patients was selected for tracing (116 from the urban clinics and 79 from the rural clinics) and 118 (60.5%) were successfully traced. 70 patients had face-to-face interviews in the clinics, 20 had telephone interviews, and 28 had home visits.

The median (IQR) age of all patients who registered in pre-ART care was 29 (24–35) years; the majority (68.2%) were females, and the median (IQR) CD4 count was 645 (529–834) cells/uL. CD4 counts were missing for 15% of patients (10% of those still in care and 27% of those who were lost); all missing data was from the rural clinics. Characteristics of patients who were still in care were generally similar to those who were lost but there was some evidence that those who were lost were most likely to be from rural clinics and to have higher CD4 counts (Table 1). Among the 195 patients who were selected for tracing, there was no evidence of a difference in the characteristics between those who were successfully traced and those who were not found. Of those who were successfully traced, 40 (33.9%) were found to be actively

For peer review only - http://bmjopen.bmj.com/site/about/guidelines.xhtml

in care (i.e. had re-registered at another clinic and were keeping up with their clinic appointments) and 71 (60.2%) were out of care. 7 (5.9%) individuals were found to have died after having left care.

At 9 months, the cumulative incidence of loss from care based on the clinic registers was 30.2% (95% confidence interval (CI)=27.3%–33.5%; Figure 1). After incorporating outcomes from those who were successfully traced, loss from care reduced to 18.5% (95%CI 13.8%–23.6%). From the sensitivity analysis, assuming that the individuals who were traced but not found were all in care then loss was 14.9% (95%CI=10.8%–19.6%). Assuming that these patients were all out of care, then loss from care increased to 38.5% (95%CI= 31.5%–45.7%). Loss from care was higher in rural than urban clinics (46.1% vs 25.8%, respectively, based on the clinic registers). When corrected for the outcomes of those who were traced, loss from care was 28.8% (95%CI=19.9%%–37.5%) in the rural clinics and 15.3% (95%CI=9.9%–21.5%) in the urban clinics.

Based on the information available from the clinic registers alone, no patients were known to have died. After tracing, 7 patients were found to have died. After incorporating the deaths that were found through tracing, the cumulative incidence of mortality at 9 months was estimated to be 1.6% (CI=0.5%–3.0%).

For peer review only

Table 1. Characteristics of patients registered for pre-ART care

Characteristics	In care (N=864) N (col %)	Lost (N=307) N (col %)	p-value[a]	Tracked (N=195) N (col %)	Not tracked (N=112) N (col %)	p-value[b]	Found (N=118) N (col %)	Not found (N=77) N (col %)	p-value[c]
Sex			0.17			0.97			0.84
Male	258 (30.5%)	109 (35.5%)		69 (35.4%)	72 (64.3%)		41 (34.7%)	28 (36.4%)	
Female	588 (69.5%)	198 (64.5%)		126 (64.6%)	40 (35.7%)		77 (65.3%)	49 (63.6%)	
Location			0.02			0.13			0.38
Urban sites	718 (84.9%)	207 (67.4%)		116 (59.5%)	91 (81.3%)		66 (55.9%)	50 (64.9%)	
Rural sites	128 (15.1%)	100 (32.6%)		79 (40.5%)	21 (18.8%)		52 (44.1%)	27 (35.1%)	
Age in years			0.15			0.25			0.44
<20	27 (3.2 %)	18 (5.9 %)		14 (7.3 %)	4 (3.6%)		10 (8.7 %)	4 (5.3 %)	
20-29	415 (49.2%)	158 (52.1%)		96 (50.3%)	62 (55.4%)		53 (46.1%)	43 (56.6%)	
30-39	266 (31.6%)	86 (28.4%)		58 (30.4%)	28 (25%)		35 (30.4%)	23 (30.3%)	
40-49	91 (10.8%)	35 (11.6%)		22 (11.5%)	13 (11.6%)		16 (13.9%)	6 (7.9 %)	
50+	44 (5.2 %)	6 (2.0 %)		1 (0.5 %)	5 (4.5%)		1 (0.9 %)	0 (0.0 %)	
Missing	3	4		4	0		3	1	
CD4 count cell			0.08			0.07			0.27
350-499	137 (18.0%)	35 (15.6%)		21 (16.5%)	14 (14.4%)		13 (17.8%)	8 (14.8%)	
500-749	376 (49.4%)	100 (44.6%)		53 (41.7%)	47 (48.5%)		34 (46.6%)	19 (35.2%)	
750+	248 (32.6%)	89 (39.7%)		53 (41.7%)	36 (37.1%)		26 (35.6%)	27 (50.0%)	
Median (IQR)	*644 (525–812)*	*654 (540–892)*		*659 (553–918)*	*648 (534–862)*		*640 (553–885)*	*734 (565–946)*	
Missing	85	83		68	15		45	23	
Weight (kg)			0.27			0.53			0.10
<50	98 (12.5%)	36 (15.4%)		19 (14.0%)	17 (17.3%)		9 (11.4%)	10 (17.5%)	
50- <60	285 (36.5%)	87 (37.2%)		48 (35.3%)	39 (39.8%)		28 (35.4%)	20 (35.1%)	
60- <70	235 (30.0%)	76 (32.5%)		45 (33.1%)	31 (31.6%)		22 (27.8%)	23 (40.4%)	
70+	164 (21.0%)	35 (15.0%)		24 (17.6%)	11 (11.2%)		20 (25.3%)	4 (7.0%)	
Median (IQR)	*60 (52–66)*	*59 (52–65)*		*60 (52–65.5)*	*58 (52–65)*		*60 (53–70)*	*59 (51–62)*	

For peer review only - http://bmjopen.bmj.com/site/about/guidelines.xhtml

Missing	64	73	59	14	39	20

[a] p value comparing those in care and lost, using Rao-Scott correction to chi squared test to account for clustered sampling. Individuals with missing values excluded from comparison. [b] p value comparing those selected for tracing and not selected, calculated as described in footnote a. [c] p value comparing those successfully traced and those not found, calculated as described in footnote a

For peer review only

In both the uncorrected and corrected analysis of factors associated with loss from care, there was strong evidence that patients from rural clinics were more likely to be lost from care than those from urban clinics (adjusted(a)HR (uncorrected)=1.95, 95%CI = 1.68–2.27, p<0.001; aHR(corrected) =2.02, 95%CI=1.49–2.73, p<0.001; Table 2). There was some evidence that older patients were less likely to be lost from care than younger patients (aHR (uncorrected) =0.79 for each 10-year increase in age, 95%CI= 0.66–0.94, p=0.007; aHR(corrected)= 0.71; 95%CI= 0.54–0.93, p=0.01). In the corrected analysis, but not in the uncorrected, there was also weak evidence that males were more likely to be lost from care.

Table 2. Factors associated with loss from care, estimated from Cox proportional hazards models, based on data in clinic registers (uncorrected) and corrected for the outcomes among patients who were successfully traced

Characteristics	Uncorrected HR[1] (95% CI)	P value	Corrected HR[1] (95% CI)	P value
Sociodemographic				
Sex		0.38		0.07
Female	1			
Male	1.17(0.82, 1.68)		1.39(0.97,1.99)	
Age per 10 years		0.007		0.01
	0.79(0.66, 0.94)		0.71(0.54,0.93)	
Location		<0.001		<0.001
Urban	1		1	
Rural	1.95(1.68, 2.27)		2.02(1.49, 2.73)	
Clinical[2]				
CD4 count (per 100 cells)		0.22		0.41
	1.04(0.98, 1.10)		1.05(0.93,1.20)	
Weight per 10kgs[3]		<0.001		<0.001
Linear term	0.94(0.80, 1.11)		1.03(0.74, 1.42)	
Quadratic term	1.03(1.02, 1.04)		0.94(0.90, 0.98)	

[1]Sociodemographic variables adjusted for all sociodemographic variables in the table. Clinical variables adjusted for all variables in the table. [2]Analysis of associations with clinical variables restricted to urban patients. [3]Weight is scaled (divided by 10) and centred on mean weight in the analysis.

For peer review only - http://bmjopen.bmj.com/site/about/guidelines.xhtml

Among patients from the urban clinics, in the uncorrected analysis, weight at registration was the only clinical characteristic associated with loss from care. Loss from care decreased with increasing weight to around 60 kilograms (kg), and then increased. After incorporating outcomes from the successfully tracked patients, weight at registration was still associated with loss from care, but the direction of the association had changed, with the risk of loss from care increasing slightly with increasing weight to around 60 kg, and then decreasing (Table 2).

Among the 71 patients who were successfully tracked and found not to be seeking care elsewhere, the main reasons for stopping care were that they lacked money for transport (37%), that they did not feel unwell (27%) or that they had moved to places without an HIV care facility (27%) (Table 3). Patients also reported that they lacked time (15%), purchased cotrimoxazole from other sources (14%) or did not believe that they were HIV positive (11%). The main reasons for stopping care among urban patients was not feeling unwell (41%) or having moved (39%). Among rural patients, the main reasons were lack of money for transport (50%) or that the clinic was too far away (43%)

Table 3. Reported reasons for leaving care or changing clinics among 111 patients who were traced and found alive

Reason for no longer attending clinic	No longer in care		
	Urban (N=41)	Rural (N=30)	All (N=71)
Lack money for transport	11 (26.8%)	15 (50.0%)	26 (36.6%)
Does not feel sick	17 (41.5%)	2 (6.7 %)	19 (26.8%)
Travelled/ moved away	16 (39.0%)	3 (10.0%)	19 (26.8%)
Health centre is far away	5 (12.2%)	13 (43.3%)	18 (25.4%)
Lack time	8 (19.5%)	3 (10.0%)	11 (15.5%)
Gets cotrimoxazole from other sources	7 (17.1%)	3 (10.0%)	10 (14.1%)
Doubts HIV status	4 (9.8 %)	4 (13.3%)	8 (11.3%)
Fear of being seen at the HIV clinic	0 (0.0 %)	5 (16.7%)	5 (7.0 %)
Does not like drugs/side effects	4 (9.8 %)	1 (3.3 %)	5 (7.0 %)
Using herbal/traditional medicines	0 (0.0 %)	3 (10.0%)	3 (4.2 %)
Other reason	3 (7.3 %)	2 (6.7 %)	5 (7.0 %)
Reason for changing clinics	In care at another clinic		
	Urban (N=23)	Rural (N=17)	All (N=40)
Closer to work	12 (52.2%)	6 (35.3%)	18 (45.0%)
Lack of money for transport	6 (26.1%)	4 (23.5%)	10 (25.0%)
Less waiting time	7 (30.4%)	0 (0.0 %)	7 (17.5%)
Lack time	5 (21.7%)	1 (5.9 %)	5 (12.5%)

For peer review only

Friends/family attend	3 (13.0%)	0 (0.0 %)	3 (7.5 %)
Fear of being seen at the first clinic	3 (13.0%)	1 (5.9 %)	3 (7.5 %)
Better service	1 (4.3 %)	1 (5.9 %)	2 (5.0 %)
Other reason	1 (4.3 %)	4 (23.5%)	5 (12.5%)

Among the 40 patients who reported being in care at another clinic, the main reasons for changing clinics was that the new clinic was closer to work or home (45%), they lacked money for transport (25%) or the new clinics had less waiting time (17%). The new clinic being closer was cited as the main reason for changing for both urban and rural patients (52% and 35%, respectively).

DISCUSSION

Much of the research that has been done regarding correction of estimates of retention in HIV care has concentrated on HIV positive individuals on ART. This study looked at individuals who had recently received an HIV diagnosis but were not yet eligible for ART. Based on the information from the clinic registers alone, loss from care was nearly 65% higher than after correcting for outcomes among individuals who were traced. We found that a third of the patients who were considered lost were continuing to access care at another clinic (silent transfers). We also identified deaths that had not been reported to the clinic. Other studies that have used a sampling based approach to correct estimates of retention among HIV positive individuals on ART have had similar findings.[12,16,17]

A study among HIV positive people in pre-ART care at two large clinics in Uganda in 2008–2011 found that loss from care after 2.5 years was 30.5% but decreased to 11.8% after correcting for outcomes in a sample of lost patients.[18] These figures are much lower than we found in our study in 2015, particularly in the rural clinics where corrected estimates of loss from care after 9 months were still 28.8%. The tracing period in our study was shorter and our definition of loss from care was more restrictive (3 months late to appointment vs 6 months late). Furthermore, some of the clinics in our study were smaller and more rural, so factors such as lack of transport or distance to the clinic may have presented greater barriers to retention. In the rural

For peer review only

areas, patients often have to travel more than 10km on foot or bicycle to get to the clinics. Lastly, the CD4 threshold for ART eligibility in the earlier study was ≤350, vs ≤500 in 2015, so a larger proportion of patients in our study may have been asymptomatic and thus less motivated to remain in care.

Our estimates of retention, even after correction, are in line with previous studies in SSA that have shown poor retention among patients in pre-ART care. A recent review found a median of 53% of patients who had linked to pre-ART care were retained until the study endpoint.[19] Even among patients who have been identified as ART eligible, a not insignificant proportion may be lost before starting ART. A study of ART-eligible patients at a clinic in Uganda found 20% did not start ART within a year, with 8% dying whilst waiting to initiate ART.[11] Two separate reviews of retention in HIV care in SSA found that around a third of patients who were eligible for ART were lost before starting treatment.[5,20]. Factors associated with loss from care in this stage include facility-level barriers such as requirements for multiple clinic visits, inflexible clinic hours, lengthy waiting times and poor quality of care, and individual-level barriers such as fear of HIV disclosure, or limited understanding of HIV.[21]

With the new WHO 'treat all' guidelines, all individuals will be eligible to start ART immediately, but in practice there is likely to be a delay between linking to care after testing positive, and initiating treatment. Removing the CD4 eligibility threshold may increase the number of patients attending the clinics, which can put a strain on already overburdened health care systems. Many of the same barriers to ART initiation will remain under 'treat all' unless the process of starting ART is made more efficient. For successful implementation of the new guidelines, it will be essential to have accurate estimates of the proportion of people who disengage from care in the period before starting ART.

In our study, most of the reported reasons for leaving care were economic (lack of money for transport, distance from the clinic) or health systems factors (moving to a location without an HIV care facility). These factors have been commonly cited in other studies, and are a challenge to providing lifelong HIV care in resource-limited settings. A systematic review of linkage to and retention in HIV care found that

For peer review only - http://bmjopen.bmj.com/site/about/guidelines.xhtml

transport costs and distance were two of the main barriers to retention in pre-ART care.[21] A considerable number of patients reported obtaining cotrimoxazole from other sources, presumably in response to the challenges they faced attending the clinic. Psychological factors such as feeling well, or not believing that one was HIV positive, were also cited as reasons for leaving care, especially among urban patients. As has been reported in other studies, we found that younger patients were more likely to be lost from care.[21] These findings suggest that a combination of interventions may be required to improve retention in care.

We used a pragmatic approach to correct our estimates of loss from care, arithmetically upweighting the outcomes of patients who were tracked successfully to represent those of patients who were lost. Other methods have been proposed for incorporating these outcomes, including using regression models to estimate the inverse probability weights, and multiple imputation in conjunction with the ascertained outcomes. Simulations have shown that these strategies all provide less biased results than the standard uncorrected approach that is used in many epidemiological studies.[22]

Our study has several limitations. We traced only a sample of patients who were lost, and were able to find 61% of those who were selected for tracing. The individuals that we found may not have been representative of all patients who were lost. Although there was no evidence that the characteristics of those who were successfully traced were different from those who were not found, our small sample size means that we may not have had power to detect true differences if they existed, and possible bias in our estimates may still remain. In addition, our sample size was based on practical considerations, rather than the power to detect a particular effect size. In addition, we looked at loss from care over a fairly short period (9 months); it is possible that some of the individuals defined as lost based on the clinic registers would have returned to the clinic at a later date. For the individuals who were successfully traced, we relied on self-report to define whether an individual was still in care at another clinic, which may have led to over-reporting of care. Our analysis of factors associated with loss from care was limited by the small number of covariates and the large amount of missing data in the clinic databases particularly from the rural areas. Our findings from government clinics in

For peer review only - http://bmjopen.bmj.com/site/about/guidelines.xhtml

Uganda may not be generalisable across all HIV treatment programmes in SSA, where reasons for disengagement from care, and outcomes after disengagement, may differ.

In summary, we found that estimates of loss from pre-ART care using a sampling based approach were substantially lower than those based on the clinic registers alone. Retention was much lower in rural clinics than in urban clinics and was in line with previous reports of pre-ART retention in SSA. Structural factors were a key barrier to retention. These findings may have implications for the successful implementation of the 'treat all' guidelines, and retention in care among individuals with high CD4 counts in similar resource-limited settings.

For peer review only

Acknowledgements

We acknowledge the Kampala City Council Authority clinic staff and officials, the Infectious Disease Institute Outreach team, and the HIV care centres in Hoima and Kabaale (Dwoli health centre III, Kigorobya health centre IV and Kagadi hospital) for their support and collaboration. We thank the study participants for their participation and the project staff for their work. We acknowledge the assistance of the Statistics unit at the Infectious Disease Institute.

Competing interests

All authors have completed the ICMJE uniform disclosure form at www.icmje.org/coi_disclosure.pdf and declare: PN and JB had research grant support from EDCTP for the submitted work; KB and JB receive research grants from MRC UK and DFID; no financial relationships with any organisations that might have an interest in the submitted work in the previous three years; no other relationships or activities that could appear to have influenced the submitted work.

Funding

This work was supported by the European & Developing Countries Clinical Trials Partnership (EDCTP) through project MF.2013.40205.020; however, EDCTP cannot accept any responsibility for information or views expressed herein. JB and KB receive support from the MRC UK and DFID (MRC grant number G0700837).

Author contribution

PN, ANK, JB and KB conceived and designed the study. PN, ANK and AK conducted the study. PN analysed the data and developed the first draft. ANK, JB and KB advised on data analysis. All authors contributed to the interpretation of the data, revised the article critically and approved the final version.

For peer review only

Data sharing statement

The original data can only be accessed by the research investigators at the Infectious Disease Institute. PN can be contacted if anonymised data or the statistical code are required

For peer review only

REFERENCES

1. World Health Organization. Progress report 2016: prevent HIV, test and treat all: WHO support for country impact. Geneva, Switzerland; 2016.
2. Stover J, Bollinger L, Izazola JA, et al., What is required to end the AIDS epidemic as a public health threat by 2030? The cost and impact of the fast-track approach. *PloS ONE* 2016;11(5):e0154893.
3. UNAIDS. 90-90-90 An ambitious treatment target to help end the AIDS epidemic. Geneva, Switzerland 2014. Available from http://www.unaids.org/en/resources/documents/2017/90-90-90.
4. Kisesa A, Chamla D. Getting to 90–90–90 targets for children and adolescents HIV in low and concentrated epidemics: bottlenecks, opportunities, and solutions. *Curr Opin HIV AIDS* 2016;11(Suppl1):S1.
5. Kranzer K, Govindasamy D, Ford N, et al. Quantifying and addressing losses along the continuum of care for people living with HIV infection in sub-Saharan Africa: a systematic review. *J Int AIDS Soc* 2012;15(2):17383
6. World Health Organization. Consolidated guidelines on the use of antiretroviral drugs for treating and preventing HIV infection. Recommendations for a public health approach. Second edition. Geneva, Switzerland; 2016.
7. Uganda AIDS Commission. Presidential Fast Track Initiative on Ending HIV and AIDS in Uganda. June 2017. http://www.aidsuganda.org/images/documents/PresidentialHandbook.pdf
8. Fox M, Rosen A. new cascade of HIV care for the era of 'treat all'. *PLoS Medicine* 2017;14(4):e1002268.
9. Geng EH, Glidden DV, Bwana MB, et al. Retention in care and connection to care among HIV-infected patients on antiretroviral therapy in Africa: estimation via a sampling-based approach. *PLoS ONE* 2011;6(7):e21797.
10. Tweya H, Feldacker C, Estill J, et al. Are they really lost? 'True' status and reasons for treatment discontinuation among HIV infected patients on antiretroviral therapy considered lost to follow up in urban Malawi. *PLoS ONE* 2013;8(9):e75761.
11. Geng EH, Bwana MB, Muyindike W, et al. Failure to initiate antiretroviral therapy, loss to follow-up and mortality among HIV-infected patients during

For peer review only

the pre-ART period in Uganda. *J Acquir Immune Defic Syndr* 2013;63(2):e64-71.

12. Geng EH, Glidden DV, Emenyonu N, et al. Tracking a sample of patients lost to follow-up has a major impact on understanding determinants of survival in HIV-infected patients on antiretroviral therapy in Africa. *Trop Med Int Health*, 2010;15(Suppl1):63-69.
13. Geng EH, Emenyonu N, Bwana MB, et al. Sampling-based approach to determining outcomes of patients lost to follow-up in antiretroviral therapy scale-up programs in Africa. *JAMA* 2008;300(5):506-507.
14. Geng EH, Odeny TA, Lyamuya R, et al. Retention in care and patient-reported reasons for undocumented transfer or stopping care among HIV-infected patients on antiretroviral therapy in Eastern Africa: application of a sampling-based approach. *Clin Infect Dis* 2015:civ1004.
15. Infectious Disease Institute College of Health Sciences Makerere University Annual Report; 2015. https://www.idi-makerere.com/index.php?option=com_docman&view=docman&Itemid=565.
16. Kiragga AN, Castelnuovo B, Musomba R, et al. Comparison of methods for correction of mortality estimates for loss to follow-up after ART initiation: a case of the Infectious Diseases Institute, Uganda. *PLoS ONE* 2013;8(12):e83524.
17. Egger M, Spycher BD, Sidle J, et al. Correcting mortality for loss to follow-up: a nomogram applied to antiretroviral treatment programmes in sub-Saharan Africa. *PLoS Med*, 2011;8(1):e1000390.
18. Namusobya J, Semitala FC, Amanyire G, et al. High retention in care among HIV-infected patients entering care with CD4 levels >350 cells/uL under routine program conditions in Uganda. *Clin Infect Dis* 2013;57(9):1343-1350.
19. Plazy M, Orne-Gliemann J, Dabis F, et al. Retention in care prior to antiretroviral treatment eligibility in sub-Saharan Africa: a systematic review of the literature. *BMJ Open* 2015;5(6):e006927.
20. Rosen S, Fox MP. Retention in HIV care between testing and treatment in sub-Saharan Africa: a systematic review. *PLoS Med* 2011;8(7):e1001056.
21. Govindasamy D, Ford N, Kranzer K. Risk factors, barriers and facilitators for linkage to antiretroviral therapy care: a systematic review. *AIDS* 2012;26(16):2059-2067.

For peer review only

22. Schomaker M, Gsponer T, Estill J, et al. Non-ignorable loss to follow-up: correcting mortality estimates based on additional outcome ascertainment. *Stat Med* 2014;33(1):129-142.

For peer review only

Figure 1: Uncorrected and corrected cumulative incidence of loss from care among patients with CD4 >500 registered for HIV care at 6 clinics

For peer review only

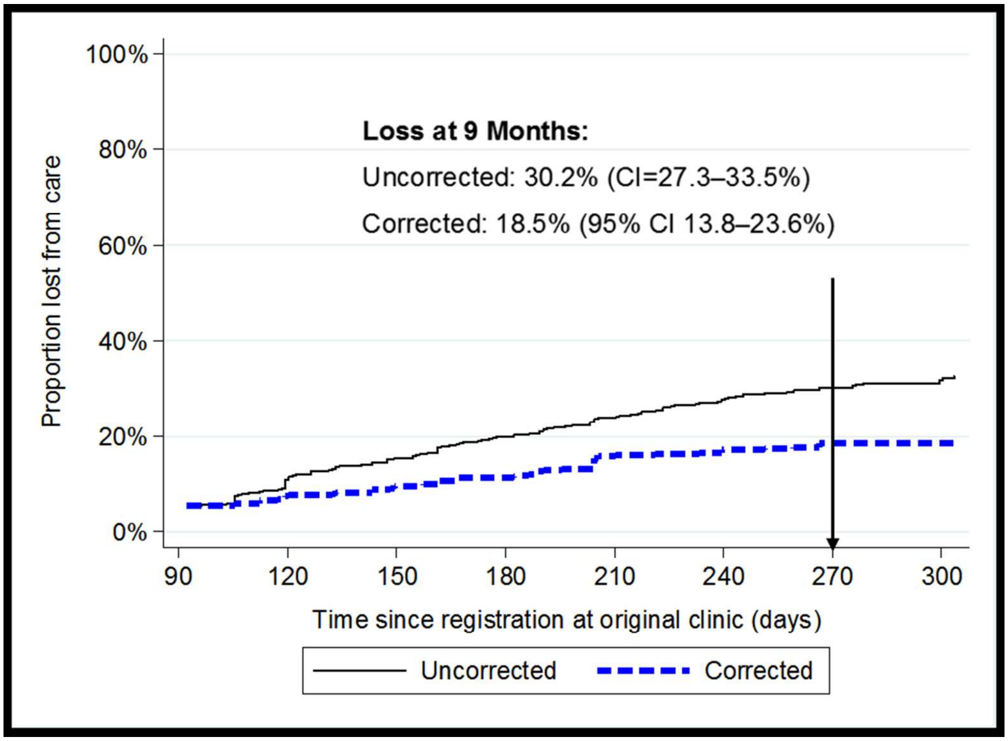

Figure 1: Uncorrected and corrected cumulative incidence of loss from care among patients with CD4 >500 registered for HIV care at 6 clinics

78x57mm (300 x 300 DPI)

STROBE 2007 (v4) checklist of items to be included in reports of observational studies in epidemiology*
Checklist for cohort, case-control, and cross-sectional studies (combined)

Section/Topic	Item #	Recommendation	Reported on page #
**Title and abstract**	1	(*a*) Indicate the study's design with a commonly used term in the title or the abstract	1
		(*b*) Provide in the abstract an informative and balanced summary of what was done and what was found	2
**Introduction**			
Background/rationale	2	Explain the scientific background and rationale for the investigation being reported	4
Objectives	3	State specific objectives, including any pre-specified hypotheses	5
**Methods**			
Study design	4	Present key elements of study design early in the paper	5, 6
Setting	5	Describe the setting, locations, and relevant dates, including periods of recruitment, exposure, follow-up, and data collection	5, 6
Participants	6	(*a*) *Cohort study*—Give the eligibility criteria, and the sources and methods of selection of participants. Describe methods of follow-up<br>*Case-control study*—Give the eligibility criteria, and the sources and methods of case ascertainment and control selection. Give the rationale for the choice of cases and controls<br>*Cross-sectional study*—Give the eligibility criteria, and the sources and methods of selection of participants	6,7
		(*b*) *Cohort study*—For matched studies, give matching criteria and number of exposed and unexposed<br>*Case-control study*—For matched studies, give matching criteria and the number of controls per case	
Variables	7	Clearly define all outcomes, exposures, predictors, potential confounders, and effect modifiers. Give diagnostic criteria, if applicable	6-8
Data sources/ measurement	8*	For each variable of interest, give sources of data and details of methods of assessment (measurement). Describe comparability of assessment methods if there is more than one group	6, 7
Bias	9	Describe any efforts to address potential sources of bias	7-8
Study size	10	Explain how the study size was arrived at	6
Quantitative variables	11	Explain how quantitative variables were handled in the analyses. If applicable, describe which groupings were chosen and why	7-8
Statistical methods	12	(*a*) Describe all statistical methods, including those used to control for confounding	7-8
		(*b*) Describe any methods used to examine subgroups and interactions	7-8
		(*c*) Explain how missing data were addressed	8
		(*d*) *Cohort study*—If applicable, explain how loss to follow-up was addressed<br>*Case-control study*—If applicable, explain how matching of cases and controls was addressed	8

For peer review only

		Cross-sectional study—If applicable, describe analytical methods taking account of sampling strategy8	
		(*e*) Describe any sensitivity analyses	8
**Results**			
Participants	13*	(a) Report numbers of individuals at each stage of study—eg numbers potentially eligible, examined for eligibility, confirmed eligible, included in the study, completing follow-up, and analysed	8-9
		(b) Give reasons for non-participation at each stage	9
		(c) Consider use of a flow diagram	N/A
Descriptive data	14*	(a) Give characteristics of study participants (eg demographic, clinical, social) and information on exposures and potential confounders	9
		(b) Indicate number of participants with missing data for each variable of interest	10
		(c) *Cohort study*—Summarise follow-up time (eg, average and total amount)	
Outcome data	15*	*Cohort study*—Report numbers of outcome events or summary measures over time	9, 11
		Case-control study—Report numbers in each exposure category, or summary measures of exposure	
		Cross-sectional study—Report numbers of outcome events or summary measures	
Main results	16	(*a*) Give unadjusted estimates and, if applicable, confounder-adjusted estimates and their precision (eg, 95% confidence interval). Make clear which confounders were adjusted for and why they were included	9, 11, 12
		(*b*) Report category boundaries when continuous variables were categorized	N/A
		(*c*) If relevant, consider translating estimates of relative risk into absolute risk for a meaningful time period	N/A
Other analyses	17	Report other analyses done—eg analyses of subgroups and interactions, and sensitivity analyses	9, 11
**Discussion**			
Key results	18	Summarise key results with reference to study objectives	13
Limitations	19	Discuss limitations of the study, taking into account sources of potential bias or imprecision. Discuss both direction and magnitude of any potential bias	15
Interpretation	20	Give a cautious overall interpretation of results considering objectives, limitations, multiplicity of analyses, results from similar studies, and other relevant evidence	15,16
Generalisability	21	Discuss the generalisability (external validity) of the study results	15
**Other information**			
Funding	22	Give the source of funding and the role of the funders for the present study and, if applicable, for the original study on which the present article is based	17

*Give information separately for cases and controls in case-control studies and, if applicable, for exposed and unexposed groups in cohort and cross-sectional studies.

Note: An Explanation and Elaboration article discusses each checklist item and gives methodological background and published examples of transparent reporting. The STROBE checklist is best used in conjunction with this article (freely available on the Web sites of PLoS Medicine at http://www.plosmedicine.org/, Annals of Internal Medicine at http://www.annals.org/, and Epidemiology at http://www.epidem.com/). Information on the STROBE Initiative is available at www.strobe-statement.org.

For peer review only

BMJ Open

Correction of estimates of retention in care among a cohort of HIV-positive patients in Uganda in the period before starting ART: a sampling-based approach

Journal:	*BMJ Open*
Manuscript ID	bmjopen-2017-017487.R2
Article Type:	Research
Date Submitted by the Author:	17-Jan-2018
Complete List of Authors:	Nyakato, Patience; London School of Hygiene and Tropical Medicine, Infectious Disease Epidemiology; Infectious Disease Institute, Makerere University<br>Kiragga, Agnes; Infectious Disease Institute, Makerere University<br>Kambugu, Andrew; Infectious Disease Institute, Makerere University<br>Bradley, John; London School of Hygiene and Tropical Medicine, MRC Tropical Epidemiology Group, Infectious Disease Epidemiology<br>Baisley, Kathy; London School of Hygiene and Tropical Medicine, MRC Tropical Epidemiology Group, Infectious Disease Epidemiology
**Primary Subject Heading**:	HIV/AIDS
Secondary Subject Heading:	Epidemiology, HIV/AIDS, Global health, Health services research
Keywords:	HIV & AIDS < INFECTIOUS DISEASES, Retention, Loss to follow up, CD4, pre-ART, Africa

SCHOLARONE™
Manuscripts

Correction of estimates of retention in care among a cohort of HIV-positive patients in Uganda in the period before starting ART: a sampling-based approach

Patience Nyakato[1,2], Agnes N Kiragga[2], Andrew Kambugu[2], John Bradley[1*], Kathy Baisley[1*]

[1]MRC Tropical Epidemiology Group, Department of Infectious Disease Epidemiology, London School of Hygiene and Tropical Medicine, London, UK; [2]Infectious Diseases Institute, College of Health Sciences, Makerere University, Kampala, Uganda

*These authors are joint senior authors on this work.

Corresponding author: Patience Nyakato; Infectious Disease Institute, Mulago Hospital Complex, College of Health Sciences, Makerere University, PO Box 22418, Kampala, Uganda. Email: pnyakato2@gmail.com Telephone: +256 787437316; Fax: +256 414 307290

Word count: 4314

For peer review only

ABSTRACT

Objective: The aim of this study was to use a sampling-based approach to obtain estimates of retention in HIV care before initiation of antiretroviral treatment (ART), corrected for outcomes in patients who were lost according to clinic registers.

Design: Retrospective cohort study of HIV positive individuals not yet eligible for ART (CD4 >500)

Setting: 3 urban and 3 rural HIV care clinics in Uganda; information was extracted from the clinic registers for all patients who had registered for pre-ART care between January–August 2015

Participants: A random sample of patients who were lost according to the clinic registers (>3 months late to scheduled visit) was traced to ascertain their outcomes.

Outcome measures: The proportion of patients lost from care was estimated using a competing risks approach, first based on the information in the clinic records alone and then using inverse probability weights to incorporate the results from tracing. Cox regression was used to determine factors associated with loss from care.

Results: Of 1153 patients registered for pre-ART care (68% female, median age 29 years, median CD4 count 645 cells/uL), 307 (27%) were lost according to clinic records. Among these, 195 (63%) were selected for tracing; outcomes were ascertained in 118 (61%). Seven patients (6%) had died, 40 (34%) were in care elsewhere, and 71 (60%) were out of care. Loss from care at 9 months was 30.2% (95% confidence interval (CI)=27.3%–33.5%). After incorporating outcomes from tracing, loss from care decreased to 18.5% (95%CI 13.8%–23.6%).

Conclusion: Estimates of loss from HIV care may be too high if based on routine clinic data alone. A sampling based approach is a feasible way of obtaining more accurate estimates of retention, accounting for transfers to other clinics.

Key words: HIV, retention in care, loss to follow up, pre-ART, CD4, Africa

For peer review only

Article Summary

Strengths and limitations of this study

- Most studies that use tracing to estimate retention in care focus on HIV-positive individuals on ART; this is one of few studies to apply these methods in the period before ART initiation.
- A sampling based approach is feasible and provides an opportunity to obtain more accurate estimates of retention in HIV care programmes in resource-limited settings
- Outcomes were not ascertained in all patients who were traced, so individuals who were traced successfully may not be representative of all who were lost.
- The follow-up time was relatively short, so some patients who were considered lost according to the clinic registers may have returned to the clinic at a later date.

For peer review only

INTRODUCTION

Access to antiretroviral therapy (ART) has expanded considerably in sub-Saharan Africa (SSA), with 12 million people in the region receiving ART in 2016.[1] With the UNAIDS 90-90-90 targets (90% of HIV positive individuals know their status, 90% of those diagnosed are on ART and 90% of those on ART are virally suppressed by 2020), more HIV positive individuals are expected to be on ART and attain viral suppression.[2-4] However, although treatment coverage in SSA doubled between 2010 and 2015, estimated coverage was still only 47% in 2015, and HIV-related mortality remains high, partly due to loss from care.[1,2]

Major gaps in HIV treatment programmes include linking individuals who test HIV positive to care, and prompt initiation of ART. Previous WHO treatment guidelines were based on CD4 count thresholds, with pre-ART care focused on immunological monitoring until individuals became eligible for ART. A 2012 systematic review of retention in HIV care in SSA found a median of 57% of individuals returned for CD4 count results after testing HIV-positive, and among those who received their results, 45% remained in care until they became eligible for ART.[5] Even among those who were ART eligible, only a median of 66% initiated ART[5].

The WHO released new HIV treatment guidelines in 2015, recommending that ART be offered to all HIV positive individuals irrespective of CD4 count.[6] If widely implemented, the 'treat all' approach would contribute significantly to achieving the 90-90-90 goals. As of end 2016, many countries in SSA had begun implementing the new guidelines. However, several countries had introduced the guidelines only in selected treatment sites, and others had not yet adopted the new policy.[1] Scale-up of ART treatment for all HIV positive people in resource-limited settings will require broad health systems strengthening, which in practice may mean that, for budgetary or other practical reasons, some clinics may still use CD4 counts to prioritise starting treatment. Uganda officially rolled out the test and treat guidelines in November 2016. By end 2017, nearly all government clinics had implemented test and treat. However, in practice, priority for ART initiation is given to existing patients in pre-ART care. Furthermore, ART 'stock outs' are still common, so individuals who

For peer review only

are newly diagnosed are likely to have some period of pre-ART care. In March 2017, an estimated 6% of HIV positive persons who were enrolled in HIV care were not on ART.[7] In addition, 48% of men who had tested HIV positive have not yet initiated ART.[7]

Under the 'treat all' guidelines, many individuals who are entering HIV care will have high CD4 counts and be asymptomatic, and therefore face different barriers to starting ART. Losses between testing HIV positive and ART initiation are still likely to remain. Obtaining accurate estimates of loss from care and outcomes in this stage will be important for evaluating the impact of the 'treat all' guidelines, and for designing interventions to improve retention and increase the numbers starting ART.[8]

Standard estimates of retention consider those who are lost to represent disengagement from care. However, many of those lost may include transfers to other care centres.[9,10] In rural Uganda, estimates of patient retention 3 years after initiating ART increased from 60% to 85% when corrected for outcomes among those who were lost.[9,11] Therefore, estimates of retention that consider patients who are lost from care to have disengaged from care are biased, and may result in misdirection of resources at the clinic and national levels.

We used a sampling-based approach to obtain more accurate estimates of loss from pre-ART care among HIV positive individuals attending clinics in Uganda who were not yet eligible for ART. This approach involves tracing a sample of lost patients and using a weighted analysis to correct the estimates of retention in the entire clinic population, with the assumption that the traced patients are representative of all who were lost.[12-14] We also explore the effect of this approach on estimates of mortality, and factors associated with loss from care before ART initiation.

For peer review only

METHODS

Study setting

This was a retrospective cohort study of patients who had registered in pre-ART HIV care at 6 government clinics in Uganda: 3 urban Kampala city municipal clinics (Kisenyi, Kawala and Kitebi) which are managed by the Kampala City Council Authority (KCCA), and 3 rural health centres in Hoima and Kibaale districts in western Uganda, which are run by the Uganda Ministry of Health. The 3 urban clinics are level IV health centres, which have a target catchment population of 100,000. Kisenyi is the largest of these, and serves a particularly economically impoverished area. The rural facilities included one level IV (Kiogorobya) and one level III (Dwoli) health centre in Hoima district, and Kagadi District Hospital in Kibaale district. Hoima district had an estimated population of 574,000 in the 2014 census, and Kibaale had an estimated population of 789,000.

All 6 facilities are supported by the Infectious Diseases Institute (IDI), Makerere University and offer integrated HIV testing and care facilities, provision of ART, and laboratory support. Most persons attend the HIV counselling and testing facilities as walk-ins. Individuals who test positive are referred for care within the clinic, or a preferred alternate facility, for immediate CD4 testing. Individuals are registered at the clinic at the time of initiating CD4 testing. They are then asked to return to the clinic within two weeks for their CD4 results. At the time of the study (2015), individuals who were not yet eligible for ART were enrolled in a general pre-ART HIV care programme, and visited the clinic every 3 months for routine clinic check-ups and cotrimoxazole prophylaxis. In 2015, the CD4 count threshold for ART initiation was ≤500 cells/uL; however, in practice, priority was given to individuals with CD4 counts <350. The 3 Kampala clinics are among 7 KCCA clinics supported by IDI; in June 2015, there were 33,514 HIV positive persons receiving HIV care services in these facilities, of whom 87% were on ART[15]

Study design

A list of all individuals who tested HIV positive between January-May 2015 (Kampala clinics) and January-August 2015 (rural clinics) and were not yet eligible for ART was

For peer review only - http://bmjopen.bmj.com/site/about/guidelines.xhtml

obtained from the routine clinic records. Socio-demographic and routine clinical data for patients attending the Kampala clinics was extracted using the electronic patient records system (OpenMRS). For patients attending the rural clinics, the information was manually extracted from the paper-based clinic records, and entered into an Access database. Patients were then classified as either i) still in care; ii) transferred out to another clinic; iii) died; or iv) lost to follow-up, based on the information available from the clinic records. Patients were counted as lost to follow-up if they were 3 or more months late for their last scheduled visit at the clinic, and not known to have transferred out or to have died.

From the sampling frame of all patients who were classified as lost, a random sample was selected, separately for urban clinics and rural clinics, for intensive tracing. The size of the sample was based on practical considerations of how many patients could be traced at each clinic with available resources, rather than on formal sample size calculations.

Tracing was done between November 2015–March 2016 in both the urban and rural clinics. Tracers attempted to contact patients through phone calls and home visits, using addresses and locator forms containing secondary phone numbers, areas of residence, and a map to the area of residence. For patients who could not be contacted through phone calls, home visits using locator forms were used; at least 3 visit attempts were made before declaring the patient unreachable.

Patients who were successfully traced were asked to provide information about their current HIV care status: whether they were registered elsewhere and were attending another clinic; whether they had started ART elsewhere; and reasons for withdrawing from HIV care if not attending any HIV care facility. Patient deaths were ascertained through an interview with a close informant.

Individuals were considered to have disengaged from care if they had not registered at any other clinic and had not returned to the clinic where they were originally registered for more than 3 months after their last scheduled visit. Individuals who said they were purchasing cotrimoxazole directly from the pharmacy (i.e. not under clinician's care) or they obtained drugs through relatives/friends were also

For peer review only

considered to have disengaged from care. Individuals who reported to have registered at another clinic were asked the name of the other clinic, the date of their next appointment, and for evidence of registration such as a patient card with a current appointment date. Those who had a current patient card, or gave a valid name of an HIV care clinic with a current appointment date, were considered to be in care.

Interviews were conducted by trained nurse counsellors, all of whom had previous research experience. Information was collected using a standard structured questionnaire. The nurse counsellors made the contact attempts by telephone, and traced urban patients at their homes if the information on the locator form was sufficient. Many of the rural patients could not be reached by telephone, and the information on the locator forms was inadequate. Therefore, in the rural clinics, ‘expert’ patients from each clinic were paired with the nurse interviewers to help trace patients in the community. Expert patients also helped with tracing in the urban clinics, for patients who could not be found through the locator forms. Expert patients serve as community volunteers at the clinic, and offer support to fellow patients in HIV care. They are familiar with the surrounding community and are often called on by the clinic to help trace persons on ART who have missed their visits.

Statistical analysis

Descriptive statistics (proportions, means, medians and interquartile ranges) were used to summarise baseline characteristics. Characteristics of patients who were retained in pre-ART care and those who were lost were tabulated and compared using Chi square tests, with the Rao-Scott correction to account for correlation within clinics. In addition, characteristics of patients who were selected for tracing and traced successfully were compared with those who were selected but could not be found.

First, using only the information available from the clinic records, the proportion of patients who were lost from pre-ART care was estimated using a cumulative incidence approach, where deaths known to the clinics were treated as a competing risk. Observation time began on the date of registration at the clinic (i.e. the date of

For peer review only - http://bmjopen.bmj.com/site/about/guidelines.xhtml

presenting for CD4 testing after HIV diagnosis) and ended at the earliest of the date of known transfer out, death, loss from care (defined as 3 months after last missed appointment) or review of the clinic records (for individuals who were still in care). Patients who initiated ART were censored on the date of ART initiation. Then, a corrected analysis was conducted using the same approach but incorporating the outcomes obtained from tracing. The outcomes of patients who were successfully traced were weighted using inverse probability weights, calculated as the inverse proportion of patients who were successfully traced among all patients who were lost. Patients who could not be found, or who were not selected for tracing, were given a weight of 0. Patients who were still in care according to the clinic registers were given a weight of 1. Weights were calculated separately for each clinic. For example, suppose that in a clinic with 100 patients of whom 30 were lost, 10 were successfully traced and 6 were found to be still in care, the weights for the patients who were traced would be 30/10=3. The corrected estimate for the proportion in care would be calculated as: [70 x 1 + 6 (found to be still in care) x 3] / [70 x 1 + 6 x 3 + 4 (found to be out of care) x 3] = 88/100. For individuals who were traced and found to still be in care, observation time was considered to end at the date of interview. Individuals who were traced and found to be alive but not in care were considered to have been lost 3 months after their last missed appointment at the original clinic.

A sensitivity analysis was also done: first we assumed that all individuals who were traced and not found were alive and in care elsewhere; second, we assumed that all patients who were not found were alive but not in care. Confidence intervals for the weighted estimates were obtained through bootstrapping using percentiles of the bootstrap distribution with 2000 replications.

Cox proportional hazards models were fitted to examine factors associated with loss from care, using data from the clinic registers alone and in a weighted analysis after incorporating results from tracing. Robust standard errors were used to account for correlation within clinics. Owing to the small number of covariates available, all variables were included in the final multivariate model. In the rural clinics, data on clinical covariates were often missing from the patient records; therefore, the analysis of clinical covariates was restricted to patients from the urban clinics. The

For peer review only - http://bmjopen.bmj.com/site/about/guidelines.xhtml

appropriate functional forms of continuous covariates were explored using low order polynomials (quadratic and cubic forms). All analyses were done using STATA version 14.2 (Stata Corp, College Station, Texas).

Ethics

Ethical approvals were obtained from the London School of Hygiene and Tropical Medicine Ethics Committee (Ref 10334), Makerere University School of Public Health Higher Degrees Research and Ethics Committee (Ref 353), Infectious Diseases Scientific Review Committee and the Uganda National Council for Science and Technology (Ref 3998). Patients give informed consent at the time of registration in HIV care at the clinics, to be traced in case they miss their appointments. Patients who were traced successfully gave additional written or oral (phone interviews) informed consent for participation in the current study.

RESULTS

Between the period of January to August 2015, 1153 individuals had registered in pre-ART care at the 6 clinics: 925 (80.2%) at the urban clinics and 228 (19.8%) at the rural clinics. 307 (26.6%) individuals were classified as lost from care (Table 1); 207 from the urban clinics (22.4% of urban patients) and 100 from the rural clinics (43.9% of rural patients). A random sample of 195 (63.5% of those lost) patients was selected for tracing (116 from the urban clinics and 79 from the rural clinics) and 118 (60.5%) were successfully traced. 70 patients had face-to-face interviews in the clinics, 20 had telephone interviews, and 28 had home visits.

The median (IQR) age of all patients who registered in pre-ART care was 29 (24–35) years; the majority (68.2%) were females, and the median (IQR) CD4 count was 645 (529–834) cells/uL. CD4 counts were missing for 15% of patients (10% of those still in care and 27% of those who were lost); all missing data was from the rural clinics. Characteristics of patients who were still in care were generally similar to those who were lost but there was some evidence that those who were lost were most likely to be from rural clinics and to have higher CD4 counts (Table 1). Among the 195 patients who were selected for tracing, there was no evidence of a difference in the characteristics between those who were successfully traced and those who were not

For peer review only

found. Of those who were successfully traced, 40 (33.9%) were found to be actively in care (i.e. had re-registered at another clinic and were keeping up with their clinic appointments) and 71 (60.2%) were out of care. 7 (5.9%) individuals were found to have died after having left care.

At 9 months, the cumulative incidence of loss from care based on the clinic registers was 30.2% (95% confidence interval (CI)=27.3%–33.5%; Figure 1). After incorporating outcomes from those who were successfully traced, loss from care reduced to 18.5% (95%CI 13.8%–23.6%). From the sensitivity analysis, assuming that the individuals who were traced but not found were all in care then loss was 14.9% (95%CI=10.8%–19.6%). Assuming that these patients were all out of care, then loss from care increased to 38.5% (95%CI= 31.5%–45.7%). Loss from care was higher in rural than urban clinics (46.1% vs 25.8%, respectively, based on the clinic registers). When corrected for the outcomes of those who were traced, loss from care was 28.8% (95%CI=19.9%%–37.5%) in the rural clinics and 15.3% (95%CI=9.9%–21.5%) in the urban clinics.

Based on the information available from the clinic registers alone, no patients were known to have died. After tracing, 7 patients were found to have died. After incorporating the deaths that were found through tracing, the cumulative incidence of mortality at 9 months was estimated to be 1.6% (CI=0.5%–3.0%).

For peer review only

Table 1. Characteristics of patients registered for pre-ART care

Characteristics	In care (N=864) N (col %)	Lost (N=307) N (col %)	p-value[a]	Tracked (N=195) N (col %)	Not tracked (N=112) N (col %)	p-value[b]	Found (N=118) N (col %)	Not found (N=77) N (col %)	p-value[c]
Sex			0.17			0.97			0.84
Male	258 (30.5%)	109 (35.5%)		69 (35.4%)	72 (64.3%)		41 (34.7%)	28 (36.4%)	
Female	588 (69.5%)	198 (64.5%)		126 (64.6%)	40 (35.7%)		77 (65.3%)	49 (63.6%)	
Location			0.02			0.13			0.38
Urban sites	718 (84.9%)	207 (67.4%)		116 (59.5%)	91 (81.3%)		66 (55.9%)	50 (64.9%)	
Rural sites	128 (15.1%)	100 (32.6%)		79 (40.5%)	21 (18.8%)		52 (44.1%)	27 (35.1%)	
Age in years			0.15			0.25			0.44
<20	27 (3.2 %)	18 (5.9 %)		14 (7.3 %)	4 (3.6%)		10 (8.7 %)	4 (5.3 %)	
20-29	415 (49.2%)	158 (52.1%)		96 (50.3%)	62 (55.4%)		53 (46.1%)	43 (56.6%)	
30-39	266 (31.6%)	86 (28.4%)		58 (30.4%)	28 (25%)		35 (30.4%)	23 (30.3%)	
40-49	91 (10.8%)	35 (11.6%)		22 (11.5%)	13 (11.6%)		16 (13.9%)	6 (7.9 %)	
50+	44 (5.2 %)	6 (2.0 %)		1 (0.5 %)	5 (4.5%)		1 (0.9 %)	0 (0.0 %)	
Missing	3	4		4	0		3	1	
CD4 count cell			0.08			0.07			0.27
350-499	137 (18.0%)	35 (15.6%)		21 (16.5%)	14 (14.4%)		13 (17.8%)	8 (14.8%)	
500-749	376 (49.4%)	100 (44.6%)		53 (41.7%)	47 (48.5%)		34 (46.6%)	19 (35.2%)	
750+	248 (32.6%)	89 (39.7%)		53 (41.7%)	36 (37.1%)		26 (35.6%)	27 (50.0%)	
Median (IQR)	*644 (525–812)*	*654 (540–892)*		*659 (553–918)*	*648 (534–862)*		*640 (553–885)*	*734 (565–946)*	
Missing	85	83		68	15		45	23	
Weight (kg)			0.27			0.53			0.10
<50	98 (12.5%)	36 (15.4%)		19 (14.0%)	17 (17.3%)		9 (11.4%)	10 (17.5%)	
50- <60	285 (36.5%)	87 (37.2%)		48 (35.3%)	39 (39.8%)		28 (35.4%)	20 (35.1%)	
60- <70	235 (30.0%)	76 (32.5%)		45 (33.1%)	31 (31.6%)		22 (27.8%)	23 (40.4%)	
70+	164 (21.0%)	35 (15.0%)		24 (17.6%)	11 (11.2%)		20 (25.3%)	4 (7.0%)	
Median (IQR)	*60 (52–66)*	*59 (52–65)*		*60 (52–65.5)*	*58 (52–65)*		*60 (53–70)*	*59 (51–62)*	

For peer review only - http://bmjopen.bmj.com/site/about/guidelines.xhtml

Missing	64	73	59	14	39	20

[a] p value comparing those in care and lost, using Rao-Scott correction to chi squared test to account for clustered sampling. Individuals with missing values excluded from comparison. [b] p value comparing those selected for tracing and not selected, calculated as described in footnote a. [c] p value comparing those successfully traced and those not found, calculated as described in footnote a

For peer review only

In both the uncorrected and corrected analysis of factors associated with loss from care, there was strong evidence that patients from rural clinics were more likely to be lost from care than those from urban clinics (adjusted(a)HR (uncorrected)=1.95, 95%CI = 1.68–2.27, p<0.001; aHR(corrected) =2.02, 95%CI=1.49–2.73, p<0.001; Table 2). There was some evidence that older patients were less likely to be lost from care than younger patients (aHR (uncorrected) =0.79 for each 10-year increase in age, 95%CI= 0.66–0.94, p=0.007; aHR(corrected)= 0.71; 95%CI= 0.54–0.93, p=0.01). In the corrected analysis, but not in the uncorrected, there was also weak evidence that males were more likely to be lost from care.

Table 2. Factors associated with loss from care, estimated from Cox proportional hazards models, based on data in clinic registers (uncorrected) and corrected for the outcomes among patients who were successfully traced

Characteristics	Uncorrected HR[1] (95% CI)	P value	Corrected HR[1] (95% CI)	P value
Sociodemographic				
Sex		0.38		0.07
Female	1			
Male	1.17(0.82, 1.68)		1.39(0.97,1.99)	
Age per 10 years		0.007		0.01
	0.79(0.66, 0.94)		0.71(0.54,0.93)	
Location		<0.001		<0.001
Urban	1		1	
Rural	1.95(1.68, 2.27)		2.02(1.49, 2.73)	
Clinical[2]				
CD4 count (per 100 cells)		0.22		0.41
	1.04(0.98, 1.10)		1.05(0.93,1.20)	
Weight per 10kgs[3]		<0.001		<0.001
Linear term	0.94(0.80, 1.11)		1.03(0.74, 1.42)	
Quadratic term	1.03(1.02, 1.04)		0.94(0.90, 0.98)	

[1]Sociodemographic variables adjusted for all sociodemographic variables in the table. Clinical variables adjusted for all variables in the table. [2]Analysis of associations with clinical variables restricted to urban patients. [3]Weight is scaled (divided by 10) and centred on mean weight in the analysis.

Among patients from the urban clinics, in the uncorrected analysis, weight at registration was the only clinical characteristic associated with loss from care. Loss from care decreased with increasing weight to around 60 kilograms (kg), and then increased. After incorporating outcomes from the successfully tracked patients, weight at registration was still associated with loss from care, but the direction of the association had changed, with the risk of loss from care increasing slightly with increasing weight to around 60 kg, and then decreasing (Table 2).

Among the 71 patients who were successfully tracked and found not to be seeking care elsewhere, the main reasons for stopping care were that they lacked money for transport (37%), that they did not feel unwell (27%) or that they had moved to places without an HIV care facility (27%) (Table 3). Patients also reported that they lacked time (15%), purchased cotrimoxazole from other sources (14%) or did not believe that they were HIV positive (11%). The main reasons for stopping care among urban patients was not feeling unwell (41%) or having moved (39%). Among rural patients, the main reasons were lack of money for transport (50%) or that the clinic was too far away (43%)

Table 3. Reported reasons for leaving care or changing clinics among 111 patients who were traced and found alive

Reason for no longer attending clinic	No longer in care		
	Urban (N=41)	Rural (N=30)	All (N=71)
Lack money for transport	11 (26.8%)	15 (50.0%)	26 (36.6%)
Does not feel sick	17 (41.5%)	2 (6.7 %)	19 (26.8%)
Travelled/ moved away	16 (39.0%)	3 (10.0%)	19 (26.8%)
Health centre is far away	5 (12.2%)	13 (43.3%)	18 (25.4%)
Lack time	8 (19.5%)	3 (10.0%)	11 (15.5%)
Gets cotrimoxazole from other sources	7 (17.1%)	3 (10.0%)	10 (14.1%)
Doubts HIV status	4 (9.8 %)	4 (13.3%)	8 (11.3%)
Fear of being seen at the HIV clinic	0 (0.0 %)	5 (16.7%)	5 (7.0 %)
Does not like drugs/side effects	4 (9.8 %)	1 (3.3 %)	5 (7.0 %)
Using herbal/traditional medicines	0 (0.0 %)	3 (10.0%)	3 (4.2 %)
Other reason	3 (7.3 %)	2 (6.7 %)	5 (7.0 %)
Reason for changing clinics	In care at another clinic		
	Urban (N=23)	Rural (N=17)	All (N=40)
Closer to work	12 (52.2%)	6 (35.3%)	18 (45.0%)
Lack of money for transport	6 (26.1%)	4 (23.5%)	10 (25.0%)
Less waiting time	7 (30.4%)	0 (0.0 %)	7 (17.5%)
Lack time	5 (21.7%)	1 (5.9 %)	5 (12.5%)

For peer review only - http://bmjopen.bmj.com/site/about/guidelines.xhtml

Friends/family attend	3 (13.0%)	0 (0.0 %)	3 (7.5 %)
Fear of being seen at the first clinic	3 (13.0%)	1 (5.9 %)	3 (7.5 %)
Better service	1 (4.3 %)	1 (5.9 %)	2 (5.0 %)
Other reason	1 (4.3 %)	4 (23.5%)	5 (12.5%)

Among the 40 patients who reported being in care at another clinic, the main reasons for changing clinics was that the new clinic was closer to work or home (45%), they lacked money for transport (25%) or the new clinics had less waiting time (17%). The new clinic being closer was cited as the main reason for changing for both urban and rural patients (52% and 35%, respectively).

DISCUSSION

Much of the research that has been done regarding correction of estimates of retention in HIV care has concentrated on HIV positive individuals on ART. This study looked at individuals who had recently received an HIV diagnosis but were not yet eligible for ART. Based on the information from the clinic registers alone, loss from care was nearly 65% higher than after correcting for outcomes among individuals who were traced. We found that a third of the patients who were considered lost were continuing to access care at another clinic (silent transfers). We also identified deaths that had not been reported to the clinic. Other studies that have used a sampling based approach to correct estimates of retention among HIV positive individuals on ART have had similar findings.[12,16,17]

A study among HIV positive people in pre-ART care at two large clinics in Uganda in 2008–2011 found that loss from care after 2.5 years was 30.5% but decreased to 11.8% after correcting for outcomes in a sample of lost patients.[18] These figures are much lower than we found in our study in 2015, particularly in the rural clinics where corrected estimates of loss from care after 9 months were still 28.8%. The tracing period in our study was shorter and our definition of loss from care was more restrictive (3 months late to appointment vs 6 months late). Furthermore, some of the clinics in our study were smaller and more rural, so factors such as lack of transport or distance to the clinic may have presented greater barriers to retention. In the rural

For peer review only

areas, patients often have to travel more than 10km on foot or bicycle to get to the clinics. Lastly, the CD4 threshold for ART eligibility in the earlier study was ≤350, vs ≤500 in 2015, so a larger proportion of patients in our study may have been asymptomatic and thus less motivated to remain in care.

Our estimates of retention, even after correction, are in line with previous studies in SSA that have shown poor retention among patients in pre-ART care. A recent review found a median of 53% of patients who had linked to pre-ART care were retained until the study endpoint.[19] Even among patients who have been identified as ART eligible, a not insignificant proportion may be lost before starting ART. A study of ART-eligible patients at a clinic in Uganda found 20% did not start ART within a year, with 8% dying whilst waiting to initiate ART.[11] Two separate reviews of retention in HIV care in SSA found that around a third of patients who were eligible for ART were lost before starting treatment.[5,20]. Factors associated with loss from care in this stage include facility-level barriers such as requirements for multiple clinic visits, inflexible clinic hours, lengthy waiting times and poor quality of care, and individual-level barriers such as fear of HIV disclosure, or limited understanding of HIV.[21]

With the new WHO 'treat all' guidelines, all individuals will be eligible to start ART immediately, but in practice there is likely to be a delay between linking to care after testing positive, and initiating treatment. Removing the CD4 eligibility threshold may increase the number of patients attending the clinics, which can put a strain on already overburdened health care systems. Many of the same barriers to ART initiation will remain under 'treat all' unless the process of starting ART is made more efficient. For successful implementation of the new guidelines, it will be essential to have accurate estimates of the proportion of people who disengage from care in the period before starting ART.

In our study, most of the reported reasons for leaving care were economic (lack of money for transport, distance from the clinic) or health systems factors (moving to a location without an HIV care facility). These factors have been commonly cited in other studies, and are a challenge to providing lifelong HIV care in resource-limited settings. A systematic review of linkage to and retention in HIV care found that

For peer review only - http://bmjopen.bmj.com/site/about/guidelines.xhtml

transport costs and distance were two of the main barriers to retention in pre-ART care.[21] A considerable number of patients reported obtaining cotrimoxazole from other sources, presumably in response to the challenges they faced attending the clinic. Psychological factors such as feeling well, or not believing that one was HIV positive, were also cited as reasons for leaving care, especially among urban patients. As has been reported in other studies, we found that younger patients were more likely to be lost from care.[21] These findings suggest that a combination of interventions may be required to improve retention in care.

We used a pragmatic approach to correct our estimates of loss from care, arithmetically upweighting the outcomes of patients who were tracked successfully to represent those of patients who were lost. Other methods have been proposed for incorporating these outcomes, including using regression models to estimate the inverse probability weights, and multiple imputation in conjunction with the ascertained outcomes. Simulations have shown that these strategies all provide less biased results than the standard uncorrected approach that is used in many epidemiological studies.[22]

Our study has several limitations. We traced only a sample of patients who were lost, and were able to find 61% of those who were selected for tracing. The individuals that we found may not have been representative of all patients who were lost. Although there was no evidence that the characteristics of those who were successfully traced were different from those who were not found, our small sample size means that we may not have had power to detect true differences if they existed, and residual selection bias may still remain. In addition, our sample size was based on practical considerations, rather than the power to detect a particular effect size. Our analysis of predictors of loss from care was underpowered to detect anything except large effects, particularly in our analysis of clinical factors which was restricted to the urban clinics. Similarly, our estimates of retention in care, and of mortality, are less precise than they would have been with a larger sample. Furthermore, there were relatively few deaths so our sample size may not have been adequate to obtain an accurate estimate of mortality. We looked at loss from care over a fairly short period (9 months); it is possible that some of the individuals defined as lost based on the clinic registers would have returned to the clinic at a

For peer review only

later date. For the individuals who were successfully traced, we relied on self-report to define whether an individual was still in care at another clinic, which may have led to over-reporting of care. Our analysis of factors associated with loss from care was limited by the small number of covariates and the large amount of missing data in the clinic databases particularly from the rural areas. Our findings from government clinics in Uganda may not be generalisable across all HIV treatment programmes in SSA, where reasons for disengagement from care, and outcomes after disengagement, may differ.

In summary, we found that estimates of loss from pre-ART care using a sampling based approach were substantially lower than those based on the clinic registers alone. Retention was much lower in rural clinics than in urban clinics and was in line with previous reports of pre-ART retention in SSA. Structural factors were a key barrier to retention. These findings may have implications for the successful implementation of the 'treat all' guidelines, and retention in care among individuals with high CD4 counts in similar resource-limited settings.

For peer review only

Acknowledgements

We acknowledge the Kampala City Council Authority clinic staff and officials, the Infectious Disease Institute Outreach team, and the HIV care centres in Hoima and Kabaale (Dwoli health centre III, Kigorobya health centre IV and Kagadi hospital) for their support and collaboration. We thank the study participants for their participation and the project staff for their work. We acknowledge the assistance of the Statistics unit at the Infectious Disease Institute.

Competing interests

All authors have completed the ICMJE uniform disclosure form at www.icmje.org/coi_disclosure.pdf and declare: PN and JB had research grant support from EDCTP for the submitted work; KB and JB receive research grants from MRC UK and DFID; no financial relationships with any organisations that might have an interest in the submitted work in the previous three years; no other relationships or activities that could appear to have influenced the submitted work.

Funding

This work was supported by the European & Developing Countries Clinical Trials Partnership (EDCTP) through project MF.2013.40205.020; however, EDCTP cannot accept any responsibility for information or views expressed herein. JB and KB receive support from the MRC UK and DFID (MRC grant number G0700837).

Author contribution

PN, ANK, JB and KB conceived and designed the study. PN, ANK and AK conducted the study. PN analysed the data and developed the first draft. ANK, JB and KB advised on data analysis. All authors contributed to the interpretation of the data, revised the article critically and approved the final version.

For peer review only

Data sharing statement

The original data can only be accessed by the research investigators at the Infectious Disease Institute. PN can be contacted if anonymised data or the statistical code are required

For peer review only

REFERENCES

1. World Health Organization. Progress report 2016: prevent HIV, test and treat all: WHO support for country impact. Geneva, Switzerland; 2016.
2. Stover J, Bollinger L, Izazola JA, et al., What is required to end the AIDS epidemic as a public health threat by 2030? The cost and impact of the fast-track approach. *PloS ONE* 2016;11(5):e0154893.
3. UNAIDS. 90-90-90 An ambitious treatment target to help end the AIDS epidemic. Geneva, Switzerland 2014. Available from http://www.unaids.org/en/resources/documents/2017/90-90-90.
4. Kisesa A, Chamla D. Getting to 90–90–90 targets for children and adolescents HIV in low and concentrated epidemics: bottlenecks, opportunities, and solutions. *Curr Opin HIV AIDS* 2016;11(Suppl1):S1.
5. Kranzer K, Govindasamy D, Ford N, et al. Quantifying and addressing losses along the continuum of care for people living with HIV infection in sub-Saharan Africa: a systematic review. *J Int AIDS Soc* 2012;15(2):17383
6. World Health Organization. Consolidated guidelines on the use of antiretroviral drugs for treating and preventing HIV infection. Recommendations for a public health approach. Second edition. Geneva, Switzerland; 2016.
7. Uganda AIDS Commission. Presidential Fast Track Initiative on Ending HIV and AIDS in Uganda. June 2017. http://www.aidsuganda.org/images/documents/PresidentialHandbook.pdf
8. Fox M, Rosen A. new cascade of HIV care for the era of 'treat all'. *PLoS Medicine* 2017;14(4):e1002268.
9. Geng EH, Glidden DV, Bwana MB, et al. Retention in care and connection to care among HIV-infected patients on antiretroviral therapy in Africa: estimation via a sampling-based approach. *PLoS ONE* 2011;6(7):e21797.
10. Tweya H, Feldacker C, Estill J, et al. Are they really lost? 'True' status and reasons for treatment discontinuation among HIV infected patients on antiretroviral therapy considered lost to follow up in urban Malawi. *PLoS ONE* 2013;8(9):e75761.
11. Geng EH, Bwana MB, Muyindike W, et al. Failure to initiate antiretroviral therapy, loss to follow-up and mortality among HIV-infected patients during

For peer review only

the pre-ART period in Uganda. *J Acquir Immune Defic Syndr* 2013;63(2):e64-71.

12. Geng EH, Glidden DV, Emenyonu N, et al. Tracking a sample of patients lost to follow-up has a major impact on understanding determinants of survival in HIV-infected patients on antiretroviral therapy in Africa. *Trop Med Int Health*, 2010;15(Suppl1):63-69.
13. Geng EH, Emenyonu N, Bwana MB, et al. Sampling-based approach to determining outcomes of patients lost to follow-up in antiretroviral therapy scale-up programs in Africa. *JAMA* 2008;300(5):506-507.
14. Geng EH, Odeny TA, Lyamuya R, et al. Retention in care and patient-reported reasons for undocumented transfer or stopping care among HIV-infected patients on antiretroviral therapy in Eastern Africa: application of a sampling-based approach. *Clin Infect Dis* 2015:civ1004.
15. Infectious Disease Institute College of Health Sciences Makerere University Annual Report; 2015. https://www.idi-makerere.com/index.php?option=com_docman&view=docman&Itemid=565.
16. Kiragga AN, Castelnuovo B, Musomba R, et al. Comparison of methods for correction of mortality estimates for loss to follow-up after ART initiation: a case of the Infectious Diseases Institute, Uganda. *PLoS ONE* 2013;8(12):e83524.
17. Egger M, Spycher BD, Sidle J, et al. Correcting mortality for loss to follow-up: a nomogram applied to antiretroviral treatment programmes in sub-Saharan Africa. *PLoS Med*, 2011;8(1):e1000390.
18. Namusobya J, Semitala FC, Amanyire G, et al. High retention in care among HIV-infected patients entering care with CD4 levels >350 cells/uL under routine program conditions in Uganda. *Clin Infect Dis* 2013;57(9):1343-1350.
19. Plazy M, Orne-Gliemann J, Dabis F, et al. Retention in care prior to antiretroviral treatment eligibility in sub-Saharan Africa: a systematic review of the literature. *BMJ Open* 2015;5(6):e006927.
20. Rosen S, Fox MP. Retention in HIV care between testing and treatment in sub-Saharan Africa: a systematic review. *PLoS Med* 2011;8(7):e1001056.
21. Govindasamy D, Ford N, Kranzer K. Risk factors, barriers and facilitators for linkage to antiretroviral therapy care: a systematic review. *AIDS* 2012;26(16):2059-2067.

For peer review only

22. Schomaker M, Gsponer T, Estill J, et al. Non-ignorable loss to follow-up: correcting mortality estimates based on additional outcome ascertainment. *Stat Med* 2014;33(1):129-142.

For peer review only

Figure 1: Uncorrected and corrected cumulative incidence of loss from care among patients with CD4 >500 registered for HIV care at 6 clinics

For peer review only

For peer review only - http://bmjopen.bmj.com/site/about/guidelines.xhtml

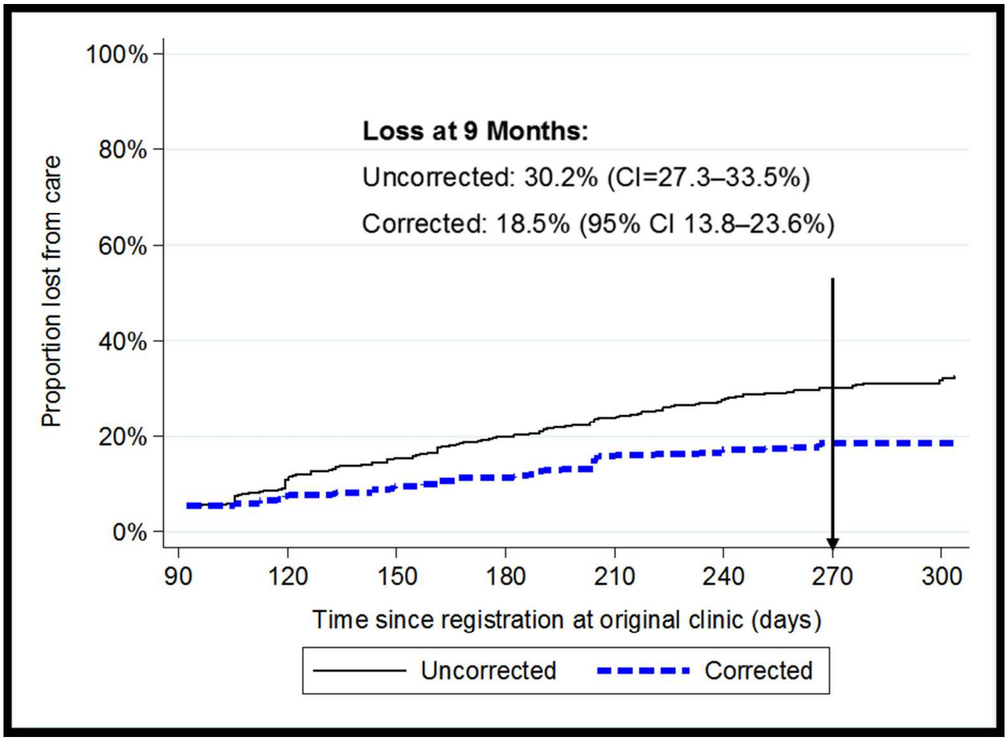

Figure 1: Uncorrected and corrected cumulative incidence of loss from care among patients with CD4 >500 registered for HIV care at 6 clinics

78x57mm (300 x 300 DPI)

STROBE 2007 (v4) checklist of items to be included in reports of observational studies in epidemiology*
Checklist for cohort, case-control, and cross-sectional studies (combined)

Section/Topic	Item #	Recommendation	Reported on page #
**Title and abstract**	1	(*a*) Indicate the study's design with a commonly used term in the title or the abstract	1
		(*b*) Provide in the abstract an informative and balanced summary of what was done and what was found	2
**Introduction**			
Background/rationale	2	Explain the scientific background and rationale for the investigation being reported	4
Objectives	3	State specific objectives, including any pre-specified hypotheses	5
**Methods**			
Study design	4	Present key elements of study design early in the paper	5, 6
Setting	5	Describe the setting, locations, and relevant dates, including periods of recruitment, exposure, follow-up, and data collection	5, 6
Participants	6	(*a*) *Cohort study*—Give the eligibility criteria, and the sources and methods of selection of participants. Describe methods of follow-up<br>*Case-control study*—Give the eligibility criteria, and the sources and methods of case ascertainment and control selection. Give the rationale for the choice of cases and controls<br>*Cross-sectional study*—Give the eligibility criteria, and the sources and methods of selection of participants	6,7
		(*b*) *Cohort study*—For matched studies, give matching criteria and number of exposed and unexposed<br>*Case-control study*—For matched studies, give matching criteria and the number of controls per case	
Variables	7	Clearly define all outcomes, exposures, predictors, potential confounders, and effect modifiers. Give diagnostic criteria, if applicable	6-8
Data sources/ measurement	8*	For each variable of interest, give sources of data and details of methods of assessment (measurement). Describe comparability of assessment methods if there is more than one group	6, 7
Bias	9	Describe any efforts to address potential sources of bias	7-8
Study size	10	Explain how the study size was arrived at	6
Quantitative variables	11	Explain how quantitative variables were handled in the analyses. If applicable, describe which groupings were chosen and why	7-8
Statistical methods	12	(*a*) Describe all statistical methods, including those used to control for confounding	7-8
		(*b*) Describe any methods used to examine subgroups and interactions	7-8
		(*c*) Explain how missing data were addressed	8
		(*d*) *Cohort study*—If applicable, explain how loss to follow-up was addressed<br>*Case-control study*—If applicable, explain how matching of cases and controls was addressed	8

For peer review only

		Cross-sectional study—If applicable, describe analytical methods taking account of sampling strategy8	
		(*e*) Describe any sensitivity analyses	8
**Results**			
Participants	13*	(a) Report numbers of individuals at each stage of study—eg numbers potentially eligible, examined for eligibility, confirmed eligible, included in the study, completing follow-up, and analysed	8-9
		(b) Give reasons for non-participation at each stage	9
		(c) Consider use of a flow diagram	N/A
Descriptive data	14*	(a) Give characteristics of study participants (eg demographic, clinical, social) and information on exposures and potential confounders	9
		(b) Indicate number of participants with missing data for each variable of interest	10
		(c) *Cohort study*—Summarise follow-up time (eg, average and total amount)	
Outcome data	15*	*Cohort study*—Report numbers of outcome events or summary measures over time	9, 11
		Case-control study—Report numbers in each exposure category, or summary measures of exposure	
		Cross-sectional study—Report numbers of outcome events or summary measures	
Main results	16	(*a*) Give unadjusted estimates and, if applicable, confounder-adjusted estimates and their precision (eg, 95% confidence interval). Make clear which confounders were adjusted for and why they were included	9, 11, 12
		(*b*) Report category boundaries when continuous variables were categorized	N/A
		(*c*) If relevant, consider translating estimates of relative risk into absolute risk for a meaningful time period	N/A
Other analyses	17	Report other analyses done—eg analyses of subgroups and interactions, and sensitivity analyses	9, 11
**Discussion**			
Key results	18	Summarise key results with reference to study objectives	13
Limitations	19	Discuss limitations of the study, taking into account sources of potential bias or imprecision. Discuss both direction and magnitude of any potential bias	15
Interpretation	20	Give a cautious overall interpretation of results considering objectives, limitations, multiplicity of analyses, results from similar studies, and other relevant evidence	15,16
Generalisability	21	Discuss the generalisability (external validity) of the study results	15
**Other information**			
Funding	22	Give the source of funding and the role of the funders for the present study and, if applicable, for the original study on which the present article is based	17

*Give information separately for cases and controls in case-control studies and, if applicable, for exposed and unexposed groups in cohort and cross-sectional studies.

Note: An Explanation and Elaboration article discusses each checklist item and gives methodological background and published examples of transparent reporting. The STROBE checklist is best used in conjunction with this article (freely available on the Web sites of PLoS Medicine at http://www.plosmedicine.org/, Annals of Internal Medicine at http://www.annals.org/, and Epidemiology at http://www.epidem.com/). Information on the STROBE Initiative is available at www.strobe-statement.org.

For peer review only
